# The mitophagy receptors BNIP3 and NIX mediate tight attachment and expansion of the isolation membrane to mitochondria

**DOI:** 10.1083/jcb.202408166

**Published:** 2025-05-13

**Authors:** Shun-ichi Yamashita, Ritsuko Arai, Hiroshi Hada, Benjamin Scott Padman, Michael Lazarou, David C. Chan, Tomotake Kanki, Satoshi Waguri

**Affiliations:** 1 Department of Cellular Physiology, Graduate School of Medical Sciences, Kyushu University, Fukuoka, Japan; 2 Department of Anatomy and Histology, Fukushima Medical University School of Medicine, Fukushima, Japan; 3 Division of Biofunctional Sciences, Department of Integrated Health Sciences, Nagoya University Graduate School of Medicine, Nagoya, Japan; 4 Telethon Kids Institute, Perth Children’s Hospital, Nedlands, Australia; 5 The University of Western Australia, Crawley, Australia; 6 Walter and Eliza Hall Institute of Medical Research, Parkville, Australia; 7 Department of Biochemistry and Molecular Biology, Biomedicine Discovery Institute, Monash University, Melbourne, Australia; 8 Department of Medical Biology, University of Melbourne, Melbourne, Australia; 9 Division of Biology and Biological Engineering, California Institute of Technology, Pasadena, CA, USA

## Abstract

BNIP3 and NIX are the main receptors for mitophagy, but their mechanisms of action remain elusive. Here, we used correlative light EM (CLEM) and electron tomography to reveal the tight attachment of isolation membranes (IMs) to mitochondrial protrusions, often connected with ER via thin tubular and/or linear structures. In BNIP3/NIX-double knockout (DKO) HeLa cells, the ULK1 complex and nascent IM formed on mitochondria, but the IM did not expand. Artificial tethering of LC3B to mitochondria induced mitophagy that was equally efficient in DKO cells and WT cells. BNIP3 and NIX accumulated at the segregated mitochondrial protrusions via binding with LC3 through their LIR motifs but did not require dimer formation. Finally, the average distance between the IM and the mitochondrial surface in receptor-mediated mitophagy was significantly smaller than that in ubiquitin-mediated mitophagy. Collectively, these results indicate that BNIP3 and NIX are required for the tight attachment and expansion of the IM along the mitochondrial surface during mitophagy.

## Teaser

The precise roles of the mitophagy receptors BNIP3 and NIX remain unclear. This study demonstrates that they are required for tight attachment and expansion of the isolation membrane along the mitochondrial surface, supporting a characteristic morphologic feature of the mitophagy.

## Introduction

Mitophagy is a type of selective autophagy that eliminates parts of mitochondria through lysosomal degradation, thus playing important roles in maintaining mitochondrial function and cellular homeostasis in mammalian cells. Recent reports have greatly advanced our understanding of the molecular mechanisms governing the recognition of targeted mitochondria by an autophagic isolation membrane (IM) (Ganley and Simonsen, 2022; Ktistakis, 2020; Montava-Garriga and Ganley, 2020; Pickles et al., 2018). A well-established mechanism utilizes the PINK1 and Parkin-mediated pathway, where outer mitochondrial membrane (OMM) proteins are ubiquitinated and subsequently targeted by the IM, using autophagy adapter proteins such as optineurin and NDP52 (Heo et al., 2015; Lazarou et al., 2015; Richter et al., 2016; Wong and Holzbaur, 2014). Another mechanism employs mitophagy receptors residing on the OMM (e.g., NIX, BNIP3, FUNDC, FKBP8, and BCL2L13), for direct recruitment of the IM (Bhujabal et al., 2017; Hanna et al., 2012; Liu et al., 2012; Murakawa et al., 2015; Schweers et al., 2007). Most examples of experimentally induced mitophagy involve mitochondrial fragmentation that precedes autophagy, allowing the IM to engulf separated small mitochondrial fragments; this process generally requires a mitochondrial fission factor: dynamin-related protein 1 (DRP1) (Rambold et al., 2011; Tanaka et al., 2010; Twig et al., 2008). However, DRP1 is not required in mitophagy induced by iron deficiency or hypoxia. Even in the absence of DRP1, a portion of a single mitochondrion was shown to be divided from its parental mitochondrion as the IM expanded along the mitochondrial surface to form a mitophagosome (Yamashita et al., 2016). Recently, we demonstrated that BNIP3 and NIX (hereafter referred to as BNIP3/NIX) are the predominant mitophagy receptors in HeLa cells and also elucidated the physiological function of mitophagy in reducing mitochondria-derived reactive oxygen species (Yamashita et al., 2024). However, precise mechanisms by which BNIP3/NIX functions as mitophagy receptors remain unclear. Therefore, in the present study, we present electron microscopic evidence that the IM is tightly attached to the mitochondrial surface and then highlight the mechanistic roles of BNIP3/NIX in the formation of mitophagosomes.

## Results

### Ultrastructure of receptor-mediated mitophagy revealed by correlative light EM

**Figure 1.**
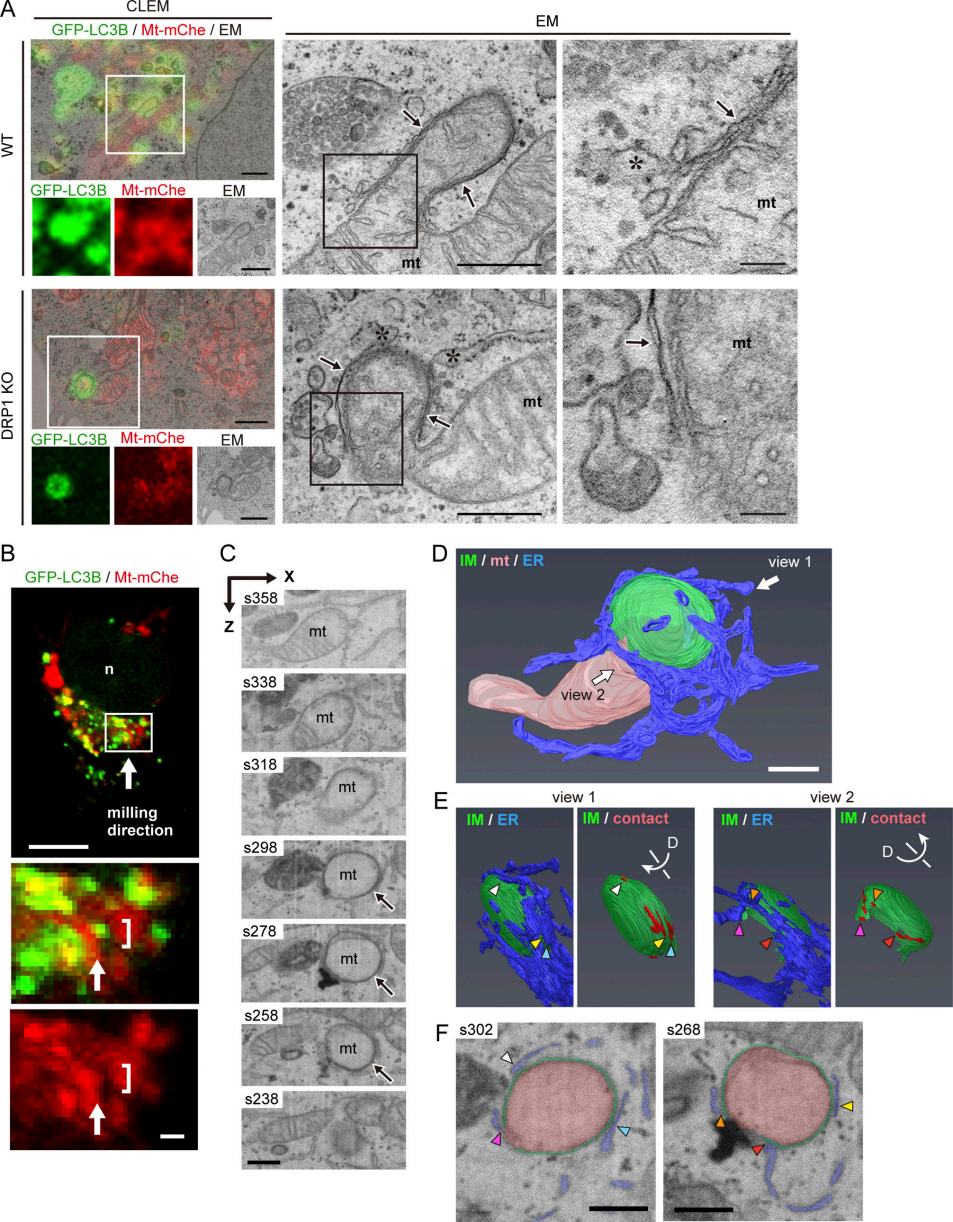
**Morphology of receptor-mediated mitophagy revealed by CLEM. (A)** Representative CLEM images (left) and enlarged EM images (right) of WT and DRP1 KO HeLa cells expressing mito-mCherry (Mt-mChe) and GFP-LC3B and treated with DFP for 16 h. Boxed areas in the main (upper) CLEM images are shown below as single-color images. Further enlargement of the boxed areas in the EM images are shown at the far right. Arrows, IM; asterisks, ER; mt, mitochondrion. Scale bars: 1 µm (CLEM), 500 nm (EM, middle), and 100 nm (EM, right). See Fig. S1 for an additional CLEM image of DRP1 KO HeLa cells. **(B–F)** Representative CLEM-FIB–scanning EM images of DRP1 KO HeLa cells expressing the same markers as in A. **(B)** Confocal image of a cell that was processed for FIB-scanning EM, with the boxed area enlarged and shown below. Arrows indicate the direction of milling. Scale bars: 10 μm (top) and 1 μm (bottom). **(C)** Serial EM images (intervals of 20 sections) taken from the bracketed region in B, represented in the x–z planes. An IM with high electron density (arrows) can be seen in section numbers 258, 278, and 298. Scale bar: 500 nm. **(D)** 3D image reconstruction from serial sections 209–408, illustrating a mitochondrion (mt, pink), ER (blue), and IM (green). **(E)** In views 1 and 2, the image from D is rotated as indicated by arrows, and the entire IM is shown with the ER (left) or IM-ER contact sites (right, red). See also Videos 1 and 2. **(F)** Two representative EM sections illustrating some of the contact sites in E (colored arrowheads). Scale bars: 500 nm.

**Figure S1.**
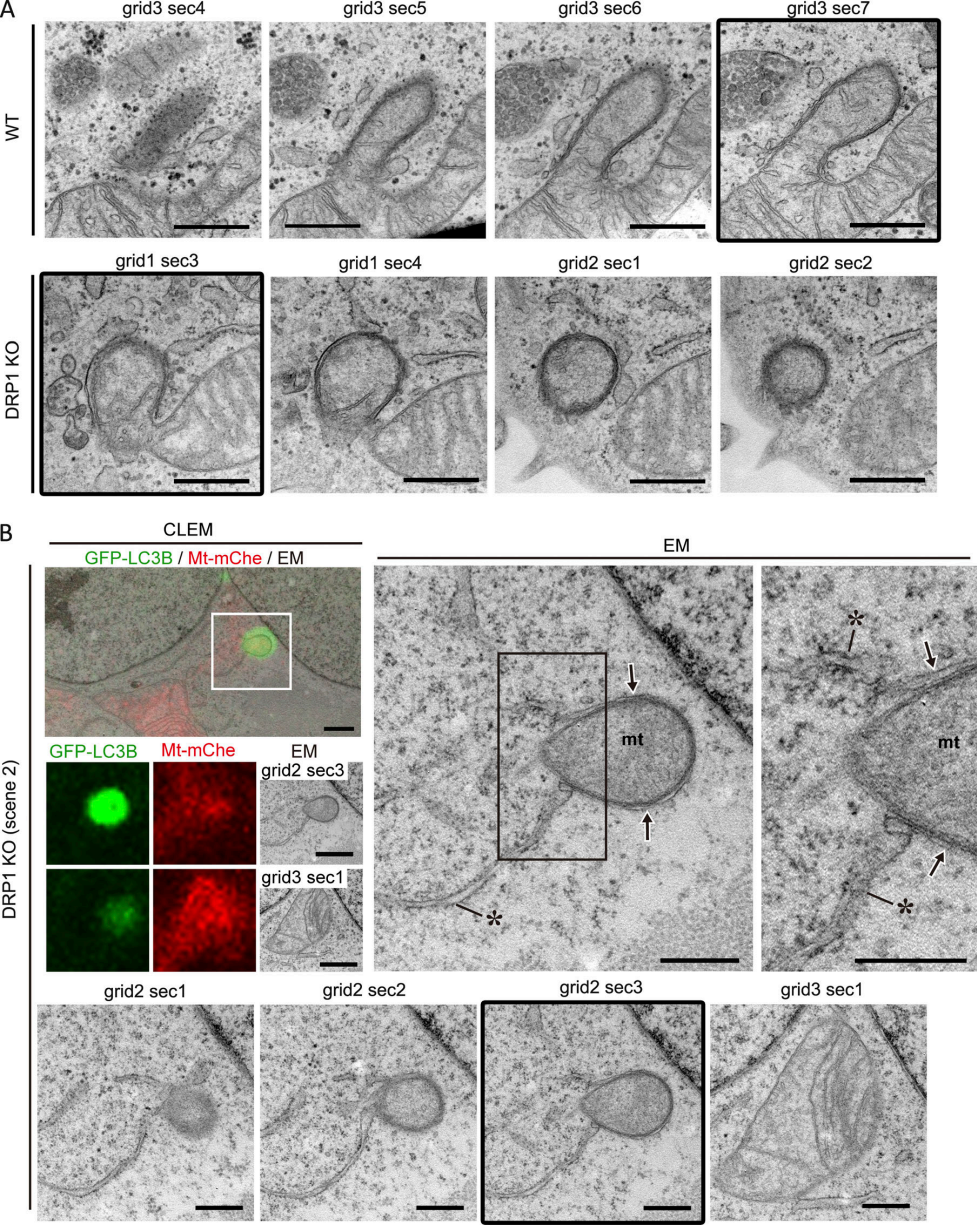
**Morphology of receptor-mediated mitophagy revealed by CLEM (related to** Fig. 1 A **). (A)** Serial ultrathin sections of WT and DRP1 KO HeLa cells, with the grid and section numbers shown above each image. The framed images appear in Fig. 1 A. **(B)** CLEM (left) of another set of serial ultrathin sections of DRP1 KO HeLa cells showing co-localization of mito-mCherry (Mt-mChe, red) and GFP-LC3B (green). The boxed area is also displayed as single-color images for GFP-LC3B, Mt-mChe, and EM below the main image. Enlargements of the grid2 sec 3 EM image (also framed in the bottom panel) are shown in the middle and right panels. EM images of serial ultrathin sections are shown in the bottom row, with the grid and section numbers shown above each. Asterisks, ER; arrows, IM; mt, mitochondrion. Scale bars: 1 µm (CLEM) and 500 nm (EM).

The kinetics of mitophagosome formation induced by the iron chelator deferiprone (DFP) has been described at the light microscopic level (Yamashita et al., 2016). To understand this process at higher spatial resolution, we used correlative light EM (CLEM) to examine the interaction of the IM with mitochondria. First, we collected EM images of the sites where GFP fused with microtubule-associated proteins 1A/1B light chain 3B (LC3B) co-localized with mito-mCherry under fluorescence microscopy. Representative images from the WT and DRP1 knockout (KO) HeLa cells are shown in Fig. 1 and Fig. S1. IMs were observed to be tightly attached to the surface of bud-like protrusions from mitochondria, covering the entire budded area. Interestingly, small vesicular structures and the ER were found near the rim of the IM, and a part of the ER appeared to be connected to the IM. To obtain larger 3D images of this type of mitophagy, we applied CLEM with focused ion beam (FIB)-scanning EM to the same groups of cells. Again, we observed the IM wrapped around a portion of a single mitochondrion, densely surrounded by ER structure (Fig. 1, B–F and Video 1). Many ER-IM contact sites were detected, accounting for ∼12.9% of the IM area and showing concentration near the IM rim, which was roughly indented (Fig. 1 E and Video 2). However, because of insufficient resolution, this experiment did not clearly reveal the detailed morphology of the contacts. These observations, together with a previous live-cell analysis (Yamashita et al., 2016), demonstrated that IMs tightly attach to protruded regions of mitochondria during DFP-induced receptor-mediated mitophagy.

**Video 1.** **Overall morphology of receptor-mediated mitophagy revealed by CLEM-FIB–scanning EM (related to** Fig. 1, C and D **).** DRP1KO HeLa cells expressing mito-mCherry and GFP-LC3 were treated with DFP for 16 h and processed for CLEM-FIB–scanning EM. In this video, serial EM images (Sections 209–408) corresponding to Fig. 1 C are shown first, followed by a color-segmented 3D reconstructed image (corresponding to Fig. 1 D) featuring a mitochondrion (pink), ER (blue), and IM (green).

**Video 2.** **Distribution of IM-ER contact sites on the IM (related to** Fig. 1 E **).** The video shows horizontal rotation of the 3D image of the IM (green) shown in Fig. 1 E, after which the IM-ER contact sites (red) are placed on it and rotate in the same manner.

### Thin tubular/linear elements connect the IM and ER in receptor-mediated mitophagy

**Figure S2.**
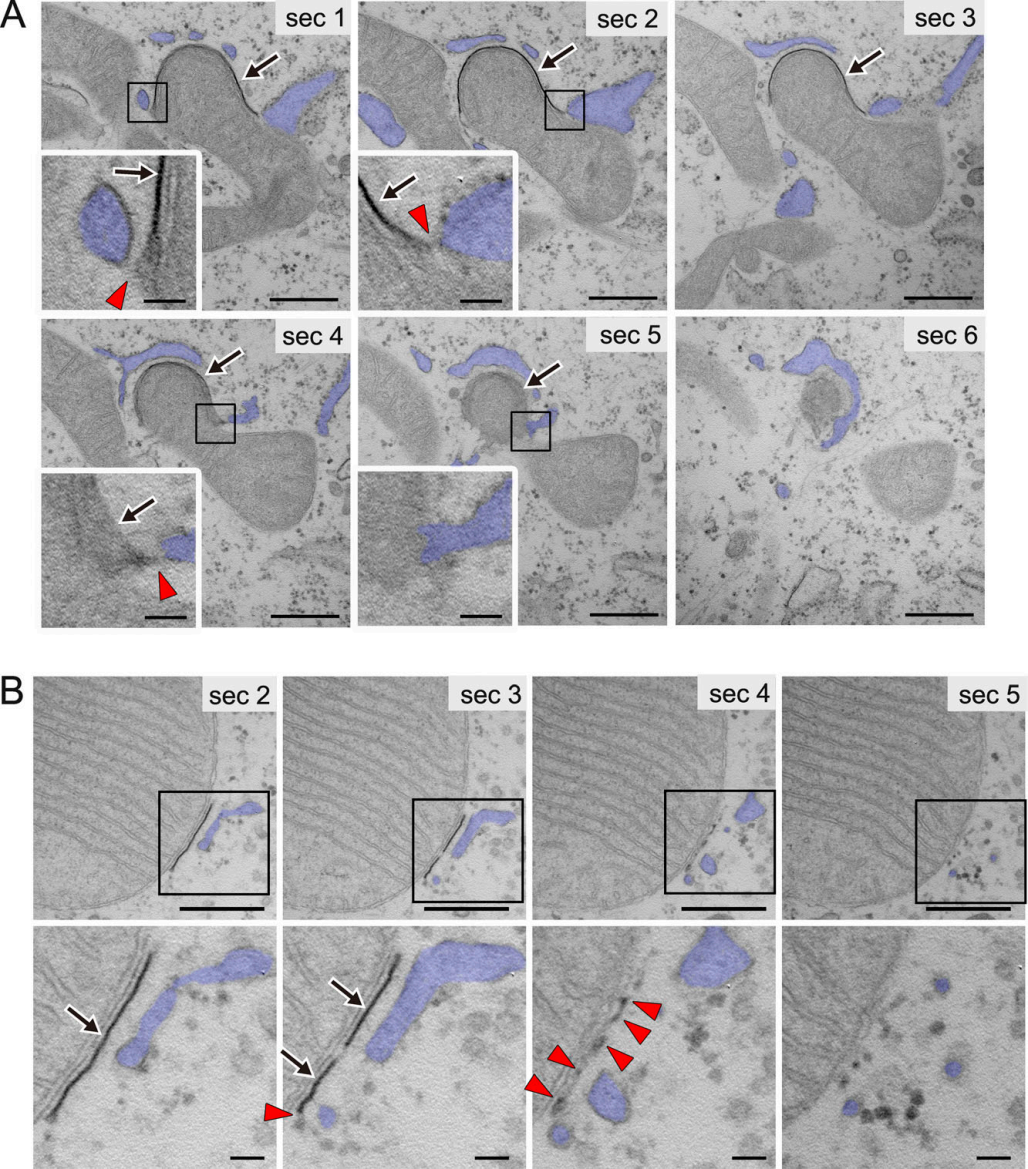
**Analysis of the fine morphology of receptor-mediated mitophagy.** Serial ultrathin sections of HeLa cells expressing mito-mCherry and GFP-LC3B, which were treated with DFP for 16 h and fixed with a mixture of aldehyde and osmium solutions. **(A and B)** Representative EM images of protruding (A) and slightly flattened (B) mitochondrial surfaces with tightly attached, electron-dense IMs (arrows). Red arrowheads indicate the frequently observed connections between ER (blue) and IM edges via thin linear/tubular structures. Boxed areas are magnified and shown in insets in A and below the main images in B. Section (sec) numbers are as indicated. Scale bars: 500 nm (lower magnification view) and 100 nm (higher magnification view).

**Figure 2.**
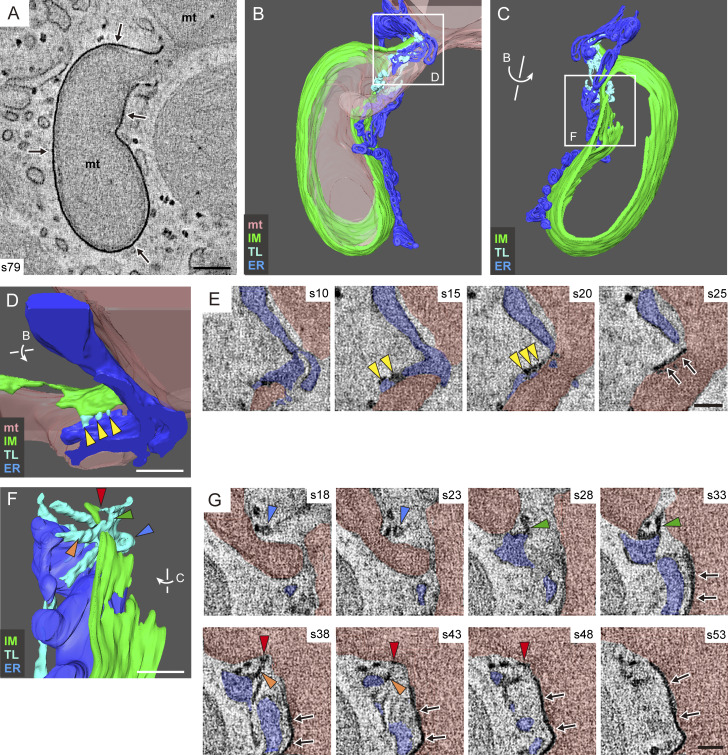
**Thin tubular/linear structures connect the IM and ER in the late phase of receptor-mediated mitophagy.** DRP1 KO HeLa cells were treated with DFP for 16 h and fixed with a mixture of aldehyde and osmium solutions for electron tomography. EM images of 102 serial sections (∼6-nm thickness) were reconstructed from two resin-embedded sections (300-nm thick each). **(A–C)** Representative EM image of section 79 (A) and its color-segmented 3D version (B) with ∼180° rotation (C), as indicated by the arrow. See also Video 3. **(D and F)** Rotated images of the boxed areas in B and C, respectively. **(E and G)** Representative EM images of the areas corresponding to D and F, respectively, in the indicated section(s) numbers. See also Videos 4 and 5. mt, mitochondria (pink); IM (green); TL, tubular/linear structures (cyan); ER (blue). Arrows in A, E, and G indicate IM. Colored arrowheads in D and F indicate tubular/linear structures, which correspond to the same-colored arrowheads in E and G, respectively. Scale bars: 200 nm (A) and 100 nm (D–G).

**Figure 3.**
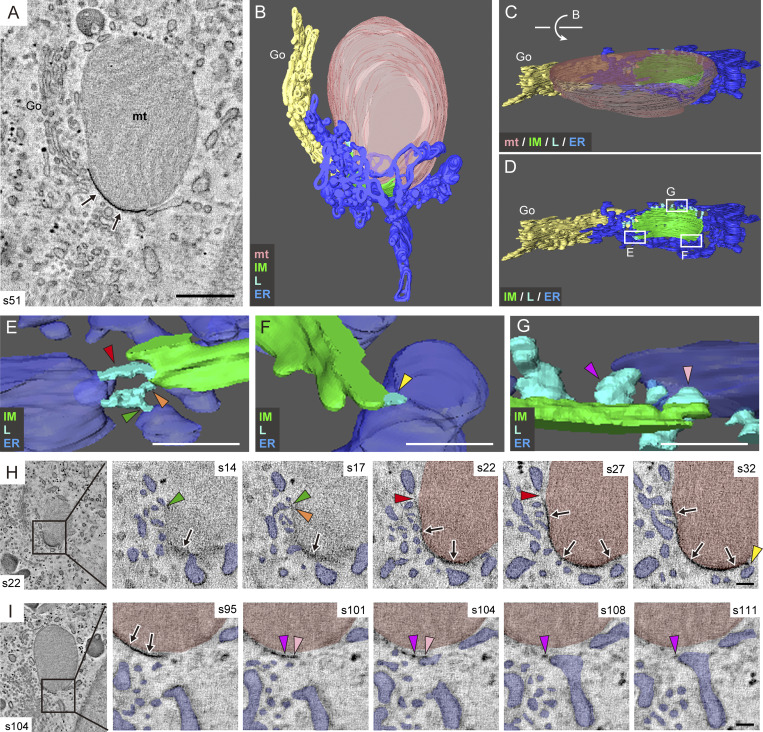
**Short linear structures connect the IM and ER in the early phase of receptor-mediated mitophagy.** DRP1 KO HeLa cells were treated with DFP for 16 h and fixed with a mixture of aldehyde and osmium solutions for electron tomography. EM images of 115 serial sections (∼6.5-nm thickness) were reconstructed from three resin-embedded sections (300-nm thick each). **(A–D)** Representative EM image of section 51 (A) and its color-segmented 3D version (B), rotated ∼100° vertically, as indicated by the arrow (C and D). See also Video 6. **(E–G)** Enlargements of the boxed and labeled areas in D, captured from slightly different angles. **(H and I)** Serial images of two representative sites indicated in the boxed areas (left), arranged from left to right and labeled with the section(s) numbers. See also Videos 7 and 8. mt (pink), mitochondria; IM (green); L, linear structure (cyan); ER (blue); Go, Golgi complex (pale yellow). Arrows in A, H, and I indicate the IM. Colored arrowheads in E–G indicate linear structures, which correspond to the same-colored arrowheads in H and I. Scale bars: 200 nm (A) and 100 nm (E–I).

To clarify the contact sites between the IM and ER, DFP-treated HeLa cells were fixed with a mixture of aldehyde and osmium fixatives to increase the preservation and electron density of the IM and IM-associated tubules (Arai and Waguri, 2019; Uemura et al., 2014). Because this fixation eliminates fluorescence signals negating the use of CLEM, we identified a mitophagy profile based only on the characteristic morphology of high-density IM tightly attached to a specific region of mitochondrial surface. DRP1 KO cells were used here because of the extremely low frequency of these profiles in WT cells, as shown in Fig. 1 A. Consistently, serial EM images demonstrated the tight association of high-density IM with the surface of the protruding region of mitochondria and the connection via tubular or linear structures between the IM rim and ER in its vicinity (Fig. S2). Interestingly, we found a region where a short high-density IM was attached to the flat, non-protruded surface of a mitochondrion, with the rim of the IM attached to tubular/linear structures (Fig. S2 B). This profile may represent an early phase of mitophagy. To corroborate these observations in 3D space, we performed electron tomography. First, we searched for profiles exhibiting the late phases of mitophagy. Fig. 2, A–C and Video 3 show a 3D model constructed from two 300-nm–thick sections. Note that the protruded region of mitochondria, nearly surrounded by a tightly attached IM, was connected to the remaining mitochondrial body through a thin neck-like region. Detailed observation revealed that the ER surrounding the neck-like region was connected to the IM via thin tubules and/or very thin linear structures (Fig. 2, D–G; and Videos 4 and 5). To observe the early phase of mitophagy, we chose an IM that was tightly attached to the flat surface of a mitochondrion (Fig. 3 A). A 3D model presented in Fig. 3, B–D and Video 6 consists of three sections, covering a total thickness of ∼900 nm and including the entire IM. The IM was surrounded by ER, which was continuous with the IM in several parts via short linear structures (Fig. 3, E–I; and Videos 7 and 8). Vesicular and tubular structures of Golgi apparatus found nearby were not directly connected to the IM. These EM analyses, including CLEM, revealed a typical morphological feature of receptor-mediated mitophagy: an ER-connected IM tightly attached to the mitochondrial surface.

**Video 3.** **Overall morphology of receptor-mediated mitophagy in the later phase revealed by electron tomography (related to** Fig. 2, A–C **).** DRP1 KO HeLa cells were treated with DFP for 16 h and fixed with a mixture of aldehyde and osmium solutions for electron tomography. In this video, serial EM images (102 sections) corresponding to Fig. 2 A are shown first, followed by color-segmented 3D reconstructed image (corresponding to Fig. 2, B and C) featuring a mitochondrion (pink), ER (blue), tubular/linear structure (cyan), and IM (green).

**Video 4.** **Linear structures connecting ER with the IM (related to** Fig. 2, D and E **).** Serial EM images (70 sections) corresponding to Fig. 2 E are shown first, followed by a color-segmented 3D reconstructed image (corresponding to Fig. 2 D) featuring a mitochondrion (pink), ER (blue), linear structures (cyan), and an IM (green).

**Video 5.** **Linear/tubular structures connecting ER with the IM (related to** Fig. 2, F and G **).** Serial EM images (53 sections) corresponding to Fig. 2 G are shown first, followed by a color-segmented 3D reconstructed image (corresponding to Fig. 2 F) featuring a mitochondrion (pink), ER (blue), linear/tubular structures (cyan), and an IM (green).

**Video 6.** **Overall morphology of receptor-mediated mitophagy in the early phase, as revealed by electron tomography (related to** Fig. 3, A–D **).** DRP1 KO HeLa cells were treated with DFP for 16 h and fixed with a mixture of aldehyde and osmium solutions for electron tomography. Serial EM images (115 sections) corresponding to Fig. 3 A are shown first, followed by color-segmented 3D reconstructed image (corresponding to Fig. 3, B–D) featuring a mitochondrion (pink), ER (blue), linear structures (cyan), an IM (green), and Golgi complex (pale yellow).

**Video 7.** **Linear structures connecting ER with the IM (related to** Fig. 3, E, F, and H **).** Serial EM images (21 sections) corresponding to Fig. 3 H are shown first, followed by a color-segmented 3D reconstructed image (corresponding to Fig. 3, E and F) featuring a mitochondrion (pink), ER (blue), linear structures (cyan), and an IM (green).

**Video 8.** **Linear structures connecting ER with the IM (related to** Fig. 3, G and I **).** Serial EM images (21 sections) corresponding to Fig. 3 I are shown first, followed by a color-segmented 3D reconstructed image (corresponding to Fig. 3 G) featuring a mitochondrion (pink), ER (blue), linear structures (cyan), and an IM (green).

### BNIP3/NIX tether the IM, but not the ULK1 complex, to mitochondria during mitophagy

**Figure S3.**
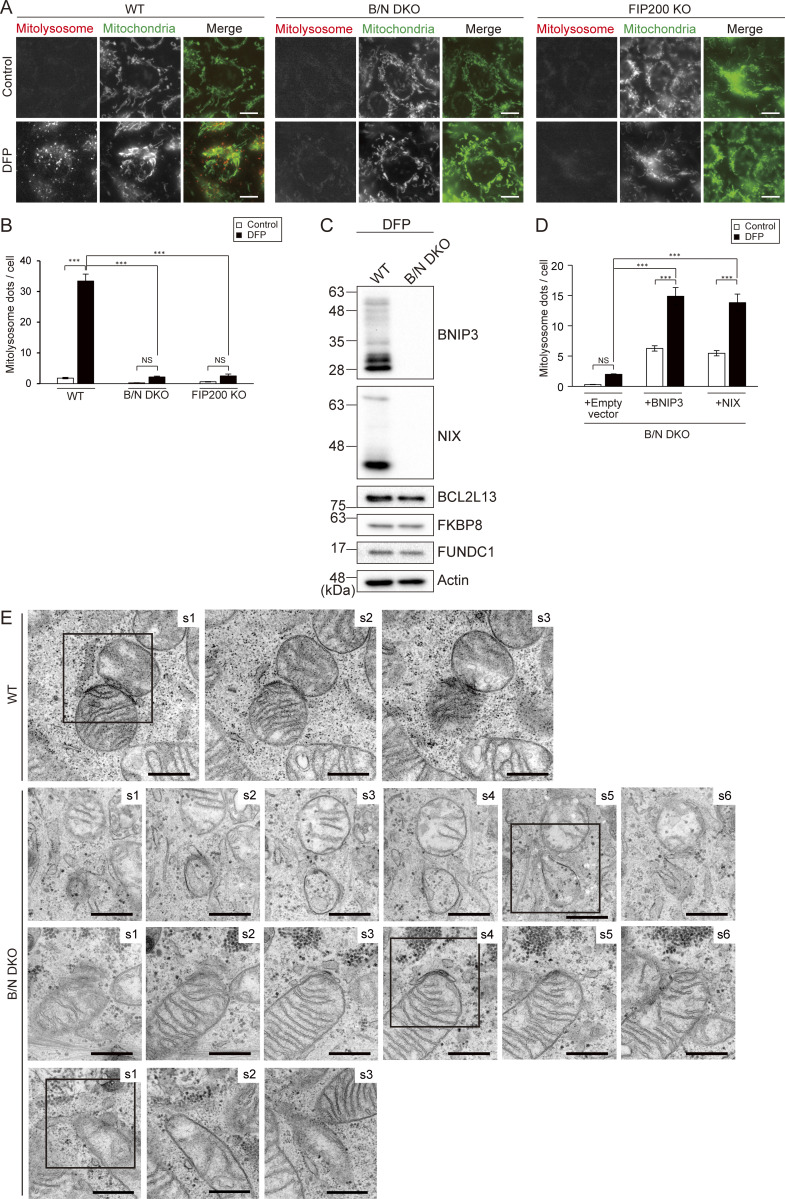
**Mitolysosome formation and recruitment of ULK1 and IM in BNIP3/NIX DKO cells. (A)** Representative images of mt-Keima in WT, BNIP3/NIX (B/N) DKO, and FIP200 KO HeLa cells, with or without DFP treatment. In the merged images, mitolysosomes (excited at 590 nm) and mitochondria (excited at 430 nm) are indicated by red and green, respectively. Scale bars: 10 μm. **(B)** Quantification of the mitolysosomes in the cells shown in A. **(C)** Immunoblot analyses of BNIP3, NIX, BCL2L13, FKBP8, and FUNDC1 in WT and B/N DKO cells under DFP treatment. **(D)** Quantification of mitolysosomes in B/N DKO cells with re-expression of BNIP3 or NIX, or the empty vector control, with or without DFP induction. Data in B and D expressed as the means ± SEM of three independent experiments involving analysis of >200 cells per experiment; ***P < 0.001 and not significant (NS), determined by one-way ANOVA followed by a Tukey–Kramer post hoc test. **(E)** Serial ultrathin sections of WT and B/N DKO HeLa cells. Section (s) numbers are indicated at the top right corner of each image. The framed images are also displayed in Fig. 5 A. Scale bars: 500 nm. Source data are available for this figure: SourceData FS3.

BNIP3/NIX are important mitophagy receptors in DFP-induced mitophagy (Hanna et al., 2012; Novak et al., 2010; Yamashita et al., 2024; Zhu et al., 2013). In fact, an assay using the fluorescence biosensor mt-Keima revealed that DFP-induced mitophagy was completely abolished in BNIP3/NIX-double KO (B/N DKO) HeLa cells generated previously by Yamashita et al. (2024) (Fig. S3, A and B). Using immunoblotting, we found no differences in the protein expression levels of three other mitophagy receptors (BCL2L13, FKBP8, and FUNDC1) between WT and B/N DKO cells under DFP treatment (Fig. S3 C), suggesting that these receptors are not involved in the mitophagy deficiency. Indeed, re-expression of BNIP3 or NIX rescued the mitophagy in B/N DKO cells (Fig. S3 D).

**Figure 4.**
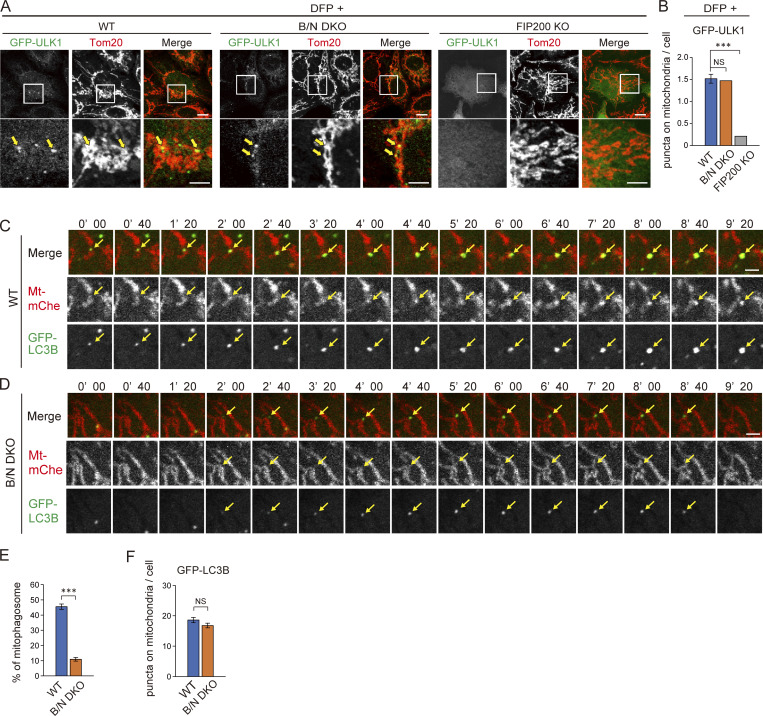
**BNIP3/NIX are required for IM elongation and mitophagosome formation. (A)** Immunofluorescence staining of Tom20 in GFP-ULK1–expressing WT, B/N DKO, and FIP200 KO HeLa cells following culture in medium containing DFP (DFP^+^) for 12 h. GFP-ULK1 puncta on mitochondria are indicated by arrows. Boxed areas are enlarged and shown below each main image. Scale bars: 10 μm (top) and 5 μm (bottom). **(B)** Quantification of the GFP-ULK1 puncta shown in A. Data are represented as the mean ± SEM (*n* = 3 biological replicates). More than 100 cells were analyzed in each replicate. **(C and D)** Time-lapse imaging of mito-mCherry (Mt-mChe) and GFP-LC3B in WT (C) and B/N DKO (D) cells cultured in DFP-containing medium. Nascent IM, elongated IM, and mitophagosomes are indicated by allows. Scale bars: 2 μm. **(E)** Quantification of the ratio of GFP-LC3B signals undergoing mitophagosome formation to the total signals shown in C and D. Data are represented as the mean ± SEM. More than 500 GFP-LC3B puncta in each cell type were analyzed. **(F)** Quantification of GFP-LC3B puncta on mitochondria shown in C and D for a duration of 30 min. Data are represented as the mean ± SEM (*n* = 30 cells). ***P < 0.001; NS, not significant by one-way ANOVA followed by Tukey–Kramer post hoc test (B) or Mann–Whitney U test (E and F).

Next, we explored the involvement of BNIP3/NIX in mitophagy. The autophagy initiation machinery, including the Unc-51–like kinase 1 (ULK1) complex, class III phosphoinositide–3-kinase complex I, and phosphoinositide-3-phosphate–binding proteins, localizes to the mitochondrial surface prior to the formation of nascent IMs during mitophagy (Itakura et al., 2012; Lazarou et al., 2015; Wu et al., 2014). To investigate whether this localization depends on BNIP3/NIX, we expressed a GFP-ULK1 fusion protein as a marker for the ULK1 complex and assessed its localization. Upon DFP treatment, GFP-ULK1 was detected as puncta on mitochondria in both WT and B/N DKO cells, but not in cells lacking FIP200, a component of the ULK1 complex (Fig. 4 A). Quantitative analysis of the mitochondrial GFP-ULK1 puncta showed no significant differences between WT and B/N DKO cells (Fig. 4 B). These results suggested that BNIP3/NIX are not required for the translocation of the autophagy initiation machineries to mitochondria.

We then examined the dynamics of IM during DFP-induced mitophagy by time-lapse imaging, visualizing mitochondria and IMs using mito-mCherry and GFP-LC3B, respectively. In WT cells, a weak GFP-LC3B punctate signal initially appeared on mitochondria, followed by an increase in the signal intensity, culminating in the separation of the corresponding mitochondrial portion from the main mitochondrial body. This suggested that the IM had emerged and elongated on the mitochondria to form mitophagosomes (Fig. 4 C), as previously reported (Yamashita et al., 2016). In contrast, B/N DKO cells produced a weak GFP-LC3B punctate signal on mitochondria, but the signal intensity did not increase, and the affected portion of mitochondria did not separate (Fig. 4 D). Quantitative analysis revealed a significant decrease in mitophagosome formation events (defined as the separation of a portion of mitochondria together with the GFP-LC3B punctate signal) in B/N DKO cells compared with that in WT cells, whereas the numbers of LC3B-positive puncta on mitochondria were comparable between the two (Fig. 4, E and F). These results suggested that BNIP3/NIX are not required for nascent IM formation on the mitochondrial surface but are essential for IM elongation and mitophagosome formation.

**Figure 5.**
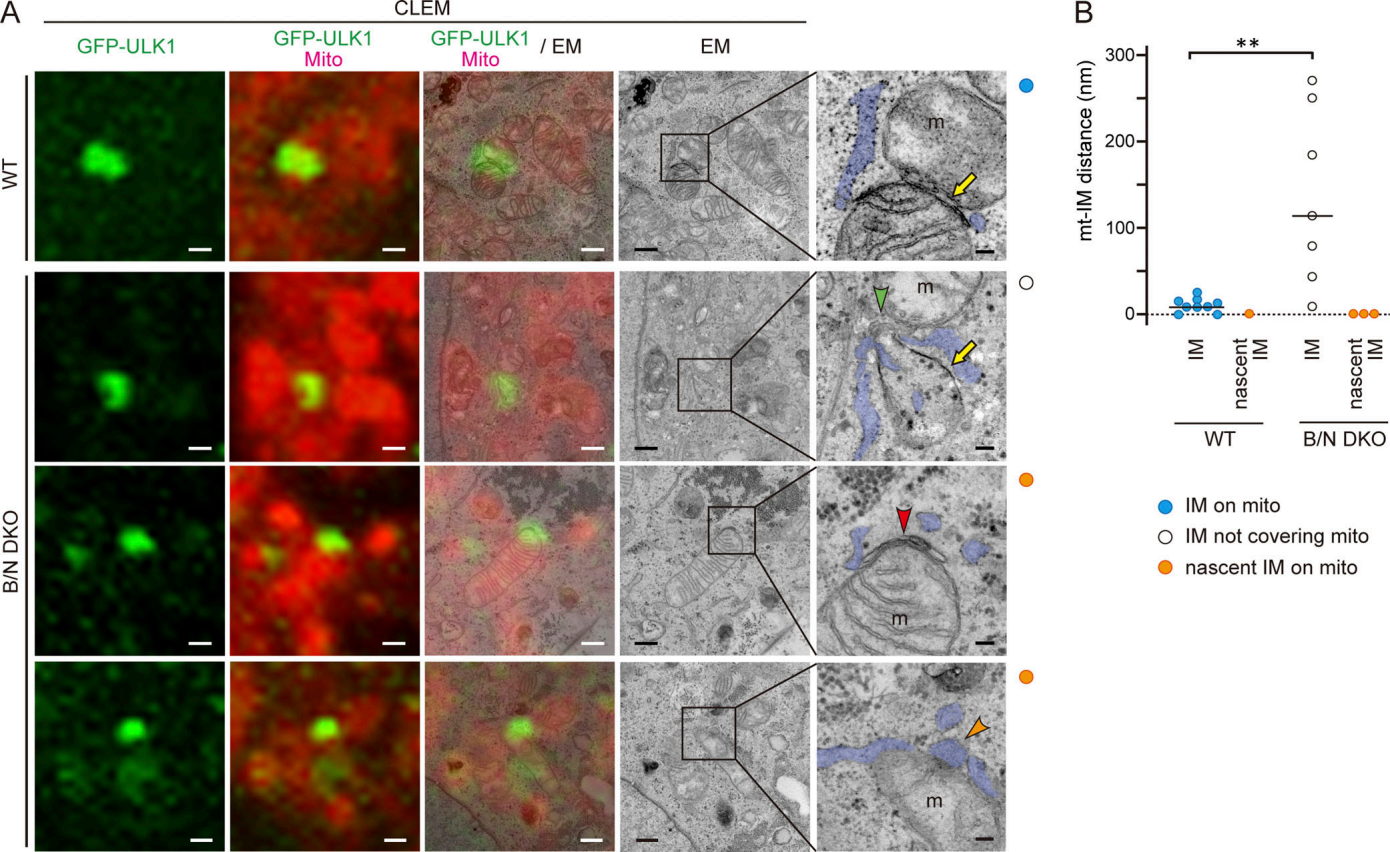
**BNIP3/NIX are required for tight attachment of the IM to the mitochondrial surface. (A)** CLEM images of MitoTracker Deep Red (Mito) and GFP-ULK1 fluorescence (left panels) and EM (right panels) of WT and B/N DKO cells expressing GFP-ULK1 and treated with DFP for 16 h. Boxed areas of EM images are enlarged and shown at the right. **(B)** Colored circles at the upper right corner outside each image correspond to the colors in the graph in B. mt, mitochondria; yellow arrow, IM; green arrowhead, IMAT; red arrowhead, IM-like structure; orange arrowhead, ER structure. Scale bars: 500 and 100 nm (EM, rightmost). **(B)** Distance between the OMM and IMs or nascent IMs in 10 profiles of WT and B/N DKO cells. EM profiles are classified into three patterns: IM attached to the mitochondrial surface (IM on mito, blue); IM not covering the mitochondrial surface (IM not covering mito, white); and IM-like structures or ER attached to the mitochondrial surface (nascent IM on mito, orange). Horizontal bars indicate the median; **P < 0.005 determined by Mann–Whitney U test. IMAT, IM-associated tubules.

To examine the fine structure of nascent IMs attached to mitochondria in B/N DKO cells, we performed CLEM of WT and B/N DKO cells expressing GFP-ULK1. Specifically, we observed 10 regions that contained GFP-ULK1–positive signals and IMs in the vicinity of mitochondria. WT cells, but not B/N DKO cells, exhibited a profile of short or long IMs tightly attached to the mitochondrial surface, showing often connection to ER in 9 out of 10 cases examined. In B/N DKO cells, IMs were present in the vicinity of mitochondria but did not cover the mitochondrial surface in 7 of the 10 cases; in the remaining three cases, short IM-like or ER structures (referred to as nascent IM) were found on the mitochondria (Fig. 5 A, and Fig. S3 E). Further, the distance between the IM and the mitochondrial surface of these 10 cases, irrespective of whether the mitochondria was covered by the IM, was significantly larger in B/N DKO cells than in WT cells (Fig. 5 B). Interestingly, nascent IMs were intimately associated with the mitochondrial surface, with no visible gap between the two. These observations suggest that ER-associated nascent IM, but not elongated typical IM, can be formed on the mitochondrial surface in B/N DKO cells. Together with the results of the time-lapse analysis, these findings indicated that BNIP3/NIX are essential for ensuring tethering of IM to the mitochondrial surface.

**Figure 6.**
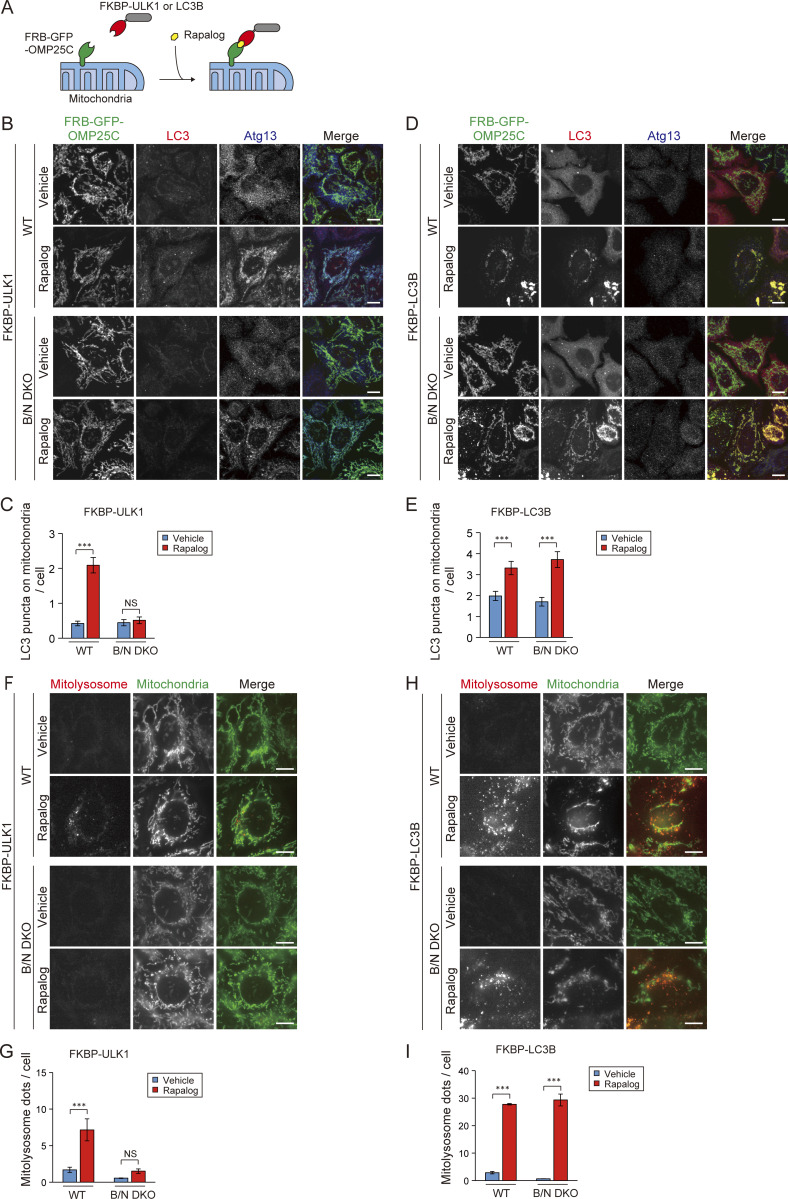
**BNIP3/NIX are required for IM tethering but not for ULK1 recruitment. (A)** Schematic representation of artificial tethering of ULK1 or LC3B to mitochondria in a chemically induced dimerization system involving treatment of cells with 500 nM rapalog for 24 h. **(B and D)** Immunofluorescence images of Atg13 and LC3 in WT and B/N DKO cells with or without artificial tethering of ULK1 (B) or LC3B (D) to mitochondria. Scale bars: 10 μm. **(C and E)** Quantification of LC3 puncta on mitochondria in the cells shown in B and D. More than 100 cells were analyzed in each group. **(F and H)** Representative images of mt-Keima in WT and B/N DKO cells with or without artificial tethering of ULK1 (F) or LC3B (H). In merged images, mitolysosomes (excited at 590 nm) and mitochondria (excited at 430 nm) are indicated by red and green, respectively. Scale bars: 10 μm. **(G and I)** Quantification of the mitolysosomes in the cells shown in F and H. Data are the averages of three independent experiments. More than 200 cells were analyzed in each experiment. Data are represented as the mean ± SEM. ***P < 0.001; NS, not significant by a Kruskal–Wallis test followed by a Steel–Dwass post hoc test (C and E) or one-way ANOVA followed by a Tukey–Kramer post hoc test (G and I).

**Figure S4.**
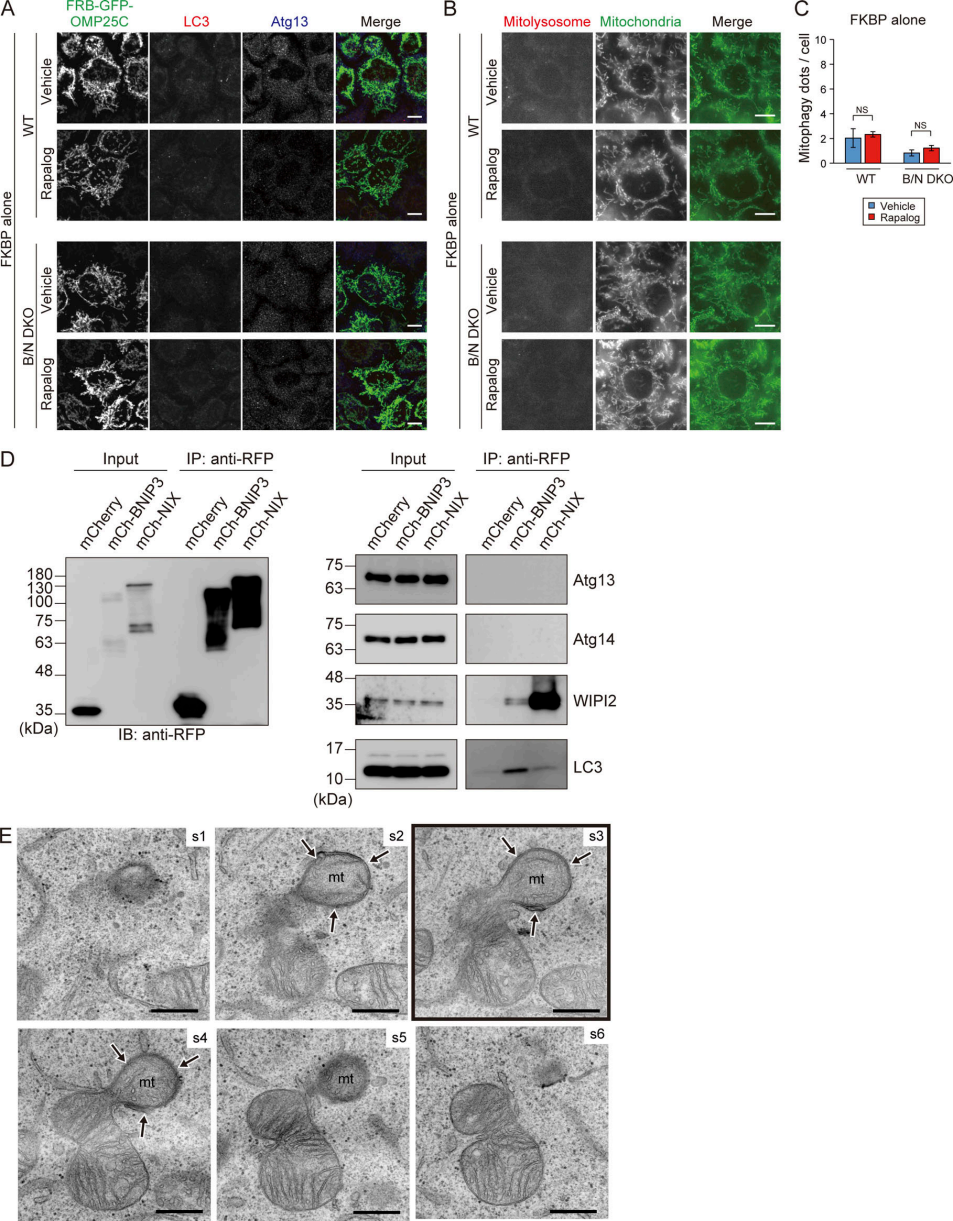
**Effects of artificial tethering of the FKBP domain, interaction analysis of BNIP3/NIX with other autophagy factors, and serial ultrathin sections related to** Fig. 7 G **. (A and B)** Representative immunofluorescence images of Atg13 and LC3 (A) and mt-Keima (B) in WT and B/N DKO cells, with or without artificial tethering of the FKBP domain to mitochondria. In merged images, mitolysosomes (excited at 590 nm) and mitochondria (excited at 430 nm) are indicated by red and green, respectively. Scale bars: 10 μm. **(C)** Quantification of the mitolysosomes in the cells shown in B. More than 200 cells were analyzed in each experiment. Data expressed as the means ± SEM of three independent experiments; ***P < 0.001 and not significant (NS), determined by one-way ANOVA followed by a Tukey–Kramer post hoc test. **(D)** Immunoprecipitation (IP) analysis of mCherry-BNIP3 or mCherry-NIX expressed in B/N DKO cells cultured with DFP for 12 h and then treated for an additional 12 h with 100 nM bafilomycin A1 and DFP. The cells were lysed, and IP was performed using RFP-trap magnetic agarose. Input is 10% of the IP fraction. **(E)** Serial ultrathin sections of the mitophagy profile. The framed image appears in Fig. 7 G. Section (s) numbers are indicated at the top right corner of each EM image. mt, mitochondrion; arrows, IM. Scale bars: 500 nm. Source data are available for this figure: SourceData FS4.

To validate our findings, we artificially tethered the ULK1 complex or IM to mitochondria using a chemically induced dimerization system (Putyrski and Schultz, 2012). First, we generated WT and B/N DKO cells stably expressing a fusion protein comprising the FKBP-rapamycin–binding domain (FRB), a GFP reporter tag, and the C terminus of human outer membrane protein 25 (OMP25C). Next, these cells were transduced with an expression vector encoding the FK506-binding protein (FKBP) domain alone or FKBP fused with ULK1 or LC3B. Upon treatment with rapalog, the ULK1 complex or the IM in these cells could be artificially tethered to mitochondrial surfaces, even in the absence of BNIP3/NIX (Fig. 6 A). When FKBP-ULK1 was tethered to mitochondria, Atg13 (a component of the ULK1 complex) was translocated to mitochondria in both WT and B/N DKO cells. Under these conditions, the number of endogenous LC3 puncta residing on mitochondria was significantly increased in WT cells but not in B/N DKO cells (Fig. 6, B and C). This is consistent with the results of time-lapse analysis (Fig. 4 D), showing that the weak signal intensity of GFP-LC3B on mitochondria was not increased by DFP inductions in B/N DKO cells. In contrast, when FKBP-LC3B was tethered to mitochondria, the signal of LC3 puncta on mitochondria increased in both WT and B/N DKO cells (Fig. 6, D and E). Next, we expressed mt-Keima in these cells and assessed mitophagy using an imaging cytometry-based assay. Consistent with the LC3 localization, mitochondrial tethering of the ULK1 complex induced mitophagy in WT cells but not in B/N DKO cells (Fig. 6, F and G), whereas tethering of LC3B-induced mitophagy to the same extent in both cell types (Fig. 6, H and I). Cells expressing FKBP alone did not exhibit translocation of the ULK1 complex or LC3B, nor did they undergo rapalog-induced mitophagy (Fig. S4, A–C). These data indicate that the recruitment of LC3B, but not ULK1, to the mitochondria is sufficient to induce mitophagy, supporting our conclusion that the primary role of BNIP3/NIX in mitophagy is to tether the IM to mitochondria.

Recent studies showing interactions between BNIP3/NIX and WD-repeat domain phosphoinositide-interacting proteins (WIPIs) suggest a novel mechanism for initiating receptor-mediated mitophagy (Adriaenssens et al., 2024, *Preprint*; Bunker et al., 2023). Therefore, we investigated the possibility that autophagy factors other than ULK1 and LC3 might associate with BNIP3 or NIX. Immunoprecipitation experiments in HeLa cells expressing mCherry-tagged BNIP3 or NIX revealed that WIPI2 interacted strongly with NIX and weakly with BNIP3, whereas Atg13 and Atg14 did not associate with either receptor (Fig. S4 D). Additionally, BNIP3 exhibited a stronger association with LC3B compared with NIX. These results suggested that BNIP3/NIX function through WIPI2 in addition to LC3.

### BNIP3/NIX assemble at mitophagosome formation sites in an LC3-interacting region-dependent manner

**Figure 7.**
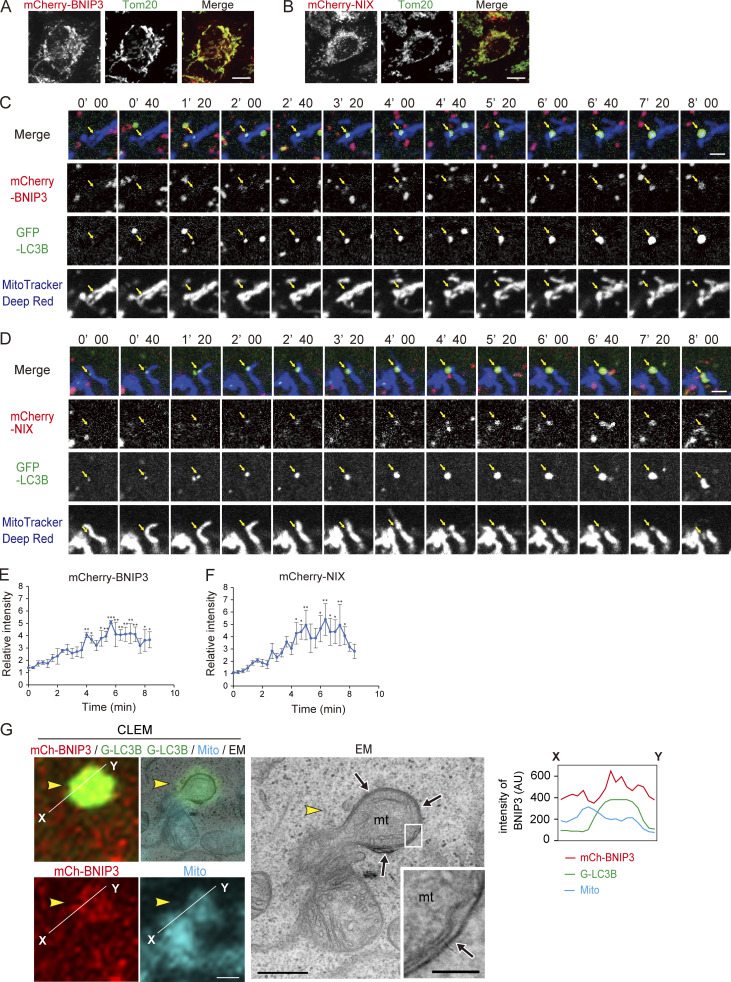
**BNIP3/NIX assemble at mitophagosome formation sites. (A and B)** Immunofluorescence images of Tom20 (green) in WT HeLa cells expressing mCherry-BNIP3 (A, red) or mCherry-NIX (B, red). Scale bars: 10 μm. **(C and D)** Time-lapse imaging of mCherry-BNIP3 (C, red) or mCherry-NIX (D, red), GFP-LC3B (green), and MitoTracker Deep Red (blue) in HeLa cells. The cells were stained with MitoTracker Deep Red prior to culturing in DFP-containing DMEM. Mitophagosome formation sites labeled by GFP-LC3B are indicated by arrows. Bars, 2 μm. **(E and F)** Quantification of fluorescence intensity of mCherry-BNIP3 (E) or mCherry-NIX (F) at mitophagosome formation sites shown in C and D. Data are represented as the mean ratio of mCherry intensity at mitophagosome formation sites to that on tubular mitochondria (which are not targeted for mitophagy) ± SEM at each time point (*n* = 3 independent events). *P < 0.05, **P < 0.01, ***P < 0.001, determined by one-way ANOVA followed by Dunnett’s post hoc test. **(G)** Representative CLEM (left) and EM (right) images of HeLa cells stably expressing mCherry-BNIP3 (mCh-BNIP3, red) and GFP-LC3B (G-LC3B, green) and cultured in DFP-containing medium for 16 h followed by staining with MitoTracker Deep Red (Mito, cyan) for 15 min. Single or overlapping images for mCh-BNIP3, G-LC3B, and mito are shown as indicated. The EM image is enlarged, with the boxed area magnified and shown as an inset. Arrowheads indicate a protruded mitochondrial region covered with IM (arrows). Fluorescence intensities of three signals along the white line in the CLEM images are plotted in the graph shown at the right. Scale bars, 500 and 100 nm (inset).

To further investigate the role of BNIP3/NIX in IM elongation on mitochondria, we performed time-lapse imaging of live cells using mCherry-tagged BNIP3/NIX, GFP-LC3B, and MitoTracker Deep Red to visualize BNIP3/NIX, IM, and mitochondria, respectively. Prior to mitophagy induction, mCherry-BNIP3 and mCherry-NIX were evenly distributed on the mitochondrial surface (Fig. 7, A and B). During mitophagy, each protein formed foci at the sites of mitophagosome formation, as labeled by GFP-LC3B puncta (Fig. 7, C and D). Notably, the intensity of these foci increased as the IM elongated (Fig. 7, E and F). Furthermore, CLEM analysis of cells stably expressing mCherry-BNIP3 showed that the intensity of the BNIP3 signal was higher in the protruded region, to which IM was attached, than in other regions (Fig. 7 G and Fig. S4 E). These data suggested that the accumulation of BNIP3/NIX promotes elongation of the IM.

**Figure S5.**
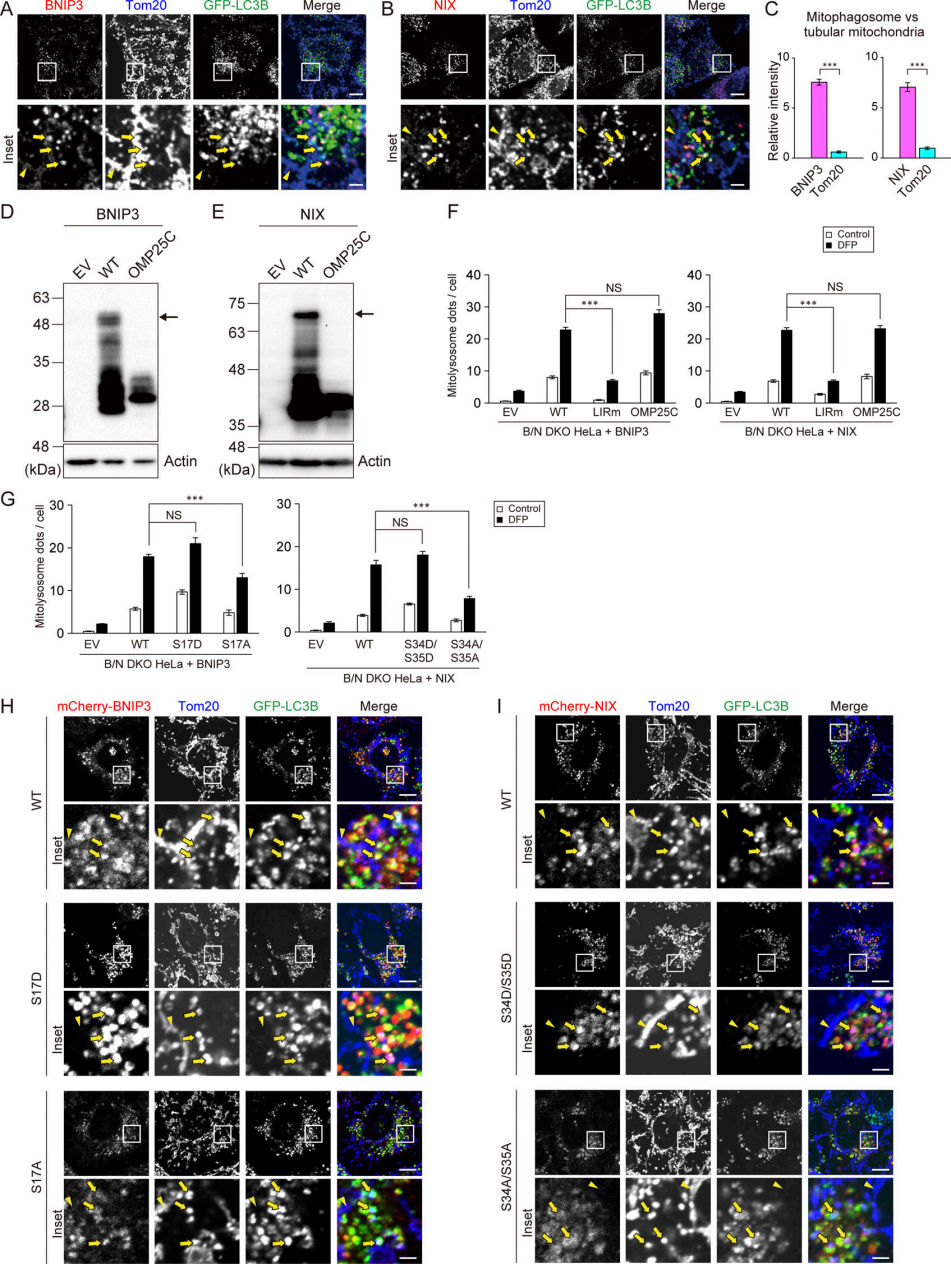
**BNIP3/NIX are accumulated in mitophagosome in an LIR-dependent manner (related to** Fig. 8 **). (A and B)** Immunofluorescence images of Tom20 (blue) and BNIP3 (A, red) or NIX (B, red) in GFP-LC3B (green)–expressing WT HeLa cells cultured in medium containing DFP for 12 h, followed by treatment with 100 nM bafilomycin A1 and DFP for an additional 12 h. Mitophagosomes (dots with positivity for both GFP-LC3B and Tom20) and tubular mitochondria are indicated by arrows and arrowheads, respectively. Scale bars: 10 μm (top) and 2 μm (bottom). **(C)** Quantification of the relative fluorescence intensities of BNIP3, NIX, and Tom20 within mitophagosomes, expressed as the mean ratio to the intensity on tubular mitochondria ± SEM (*n* = 10 cells, with >10 mitophagosomes analyzed per cell); ***P < 0.001, determined by Student’s *t* test. **(D and E)** Immunoblot analysis of WT or OMP25C hybrids of BNIP3 (D) and NIX (E) expressed in B/N DKO cells. **(F)** Quantification of the mitolysosomes in the B/N DKO cells expressing BNIP3/NIX variants. **(G)** Quantification of mitolysosomes in the B/N DKO cells expressing BNIP3/NIX serine mutants. Data in F and G represent the averages of three independent experiments involving analysis of >200 cells per experiment; ***P < 0.001, determined by a Kruskal–Wallis test followed by a Steel–Dwass post hoc test (F), or one-way ANOVA followed by Tukey–Kramer post hoc test (G). **(H and I)** Immunofluorescence images of serine mutants of mCherry-BNIP3 serine variants (H, red) or mCherry-NIX (I, red), GFP-LC3B (green), and Tom20 (blue), related to Fig. 8, G and H. Images were analyzed as in Fig. 8 A. Scale bars: 10 μm (top) and 2 μm (bottom). EV, empty vector. Source data are available for this figure: SourceData FS5.

Next, we sought to determine whether the accumulated BNIP3/NIX proteins were enclosed in the mitophagosomes. As we previously reported, mitophagosomes accumulate in the cytoplasm of cells treated with bafilomycin A1, an inhibitor of mitophagosome-lysosome fusion (Yamashita et al., 2016). Therefore, GFP-LC3B–expressing cells were cultured in DFP-containing medium in the presence of bafilomycin A1 and subjected to immunofluorescence microscopy with antibodies against BNIP3/NIX and a non-receptor mitochondrial protein, Tom20. Mitophagosomes labeled with both GFP-LC3B and Tom20 were observed in the cytoplasm and were also positive for BNIP3/NIX. Notably, the intensity of BNIP3/NIX, but not Tom20, was much higher within mitophagosomes than on the surface of tubular mitochondria, which are not a target of mitophagy (Fig. S5, A–C). This finding indicated that the accumulated BNIP3/NIX proteins are enclosed in mitophagosomes.

**Figure 8.**
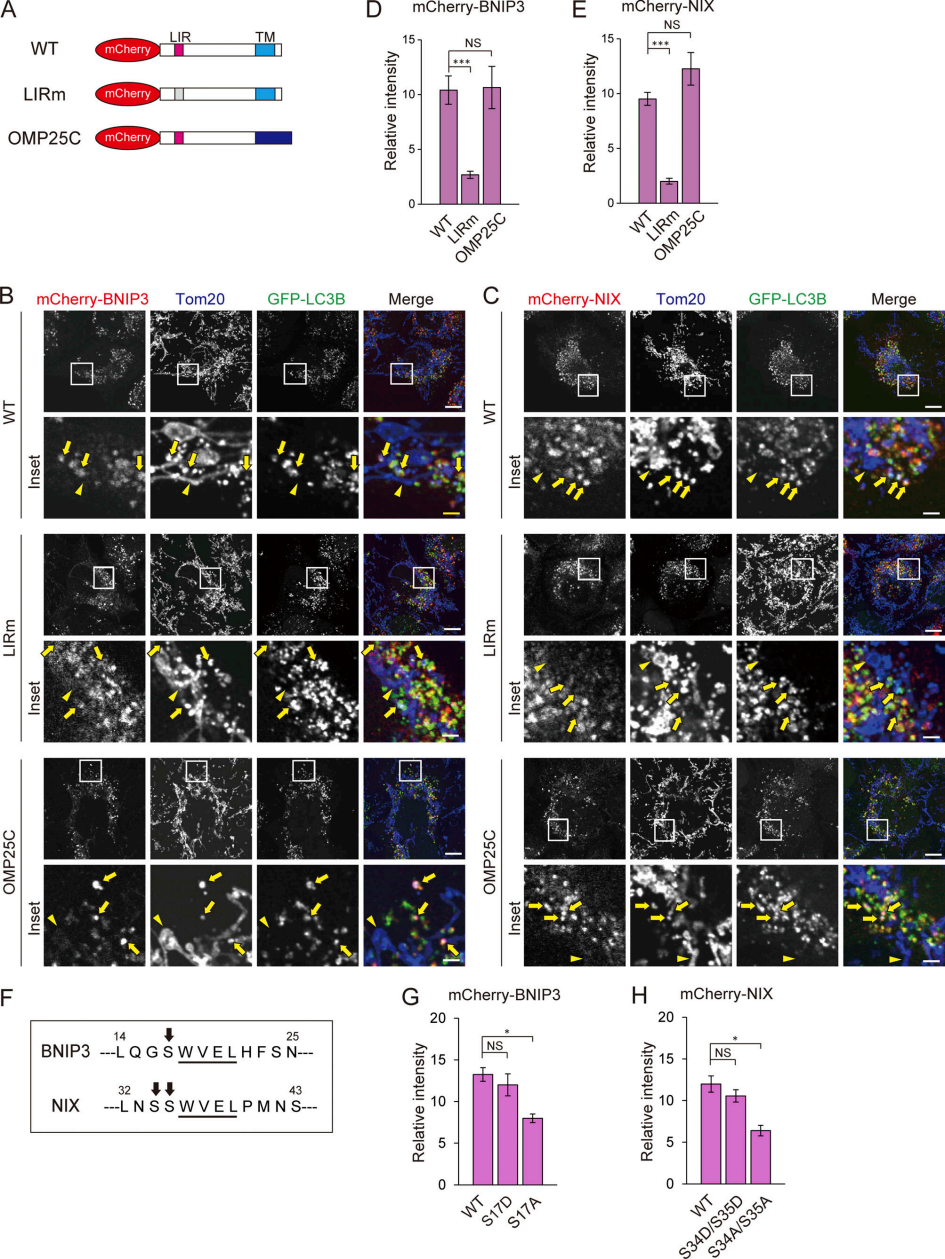
**BNIP3/NIX assemble at mitophagosome formation sites in an LIR-dependent manner. (A)** Schematic representation of BNIP3/NIX mutant constructs, showing the LIR and TM domains in the WT control, LIRm, and dimerization-deficient OMP25C mutant proteins. **(B and C)** Immunofluorescence images of mCherry-BNIP3 variants (B, red) and mCherry-NIX variants (C, red), along with GFP-LC3B (green) and Tom20 (blue) in HeLa cells cultured in DFP-containing medium for 12 h, followed by treatment with 100 nM bafilomycin A1 and DFP for an additional 12 h. Mitophagosomes and tubular mitochondria are indicated by arrows and arrowheads, respectively. Scale bars: 10 μm (top) and 2 μm (bottom). **(D and E)** Quantification of the relative fluorescence intensity of mCherry-BNIP3 or mCherry-NIX variants within mitophagosomes, represented as the mean ratio to that on tubular mitochondria ± SEM (*n* = 10 cells). More than 10 mitophagosomes were analyzed in each cell. **(F)** Serine residues (arrows) located in the vicinity of the LIRs (underlined) in BNIP3/NIX. **(G and H)** Quantification of the relative fluorescence intensity of mCherry-BNIP3 or mCherry-NIX serine mutants within mitophagosomes, as described above. Data are represented as the mean ± SEM (*n* = 6 cells). More than 10 mitophagosomes were analyzed in each cell. *P < 0.05; ***P < 0.001; NS, not significant by a Kruskal–Wallis test followed by a Steel–Dwass post hoc test (D, E, G, and H).

During mitophagy, BNIP3/NIX bind to LC3 via LC3-interacting region (LIR) motifs that are exposed to the cytoplasm tethering the IM to the mitochondrial surface. BNIP3/NIX also reportedly form homodimers via the glycine-zipper motif within transmembrane (TM) domains, enhancing mitophagy (Hanna et al., 2012; Marinkovic et al., 2021). Therefore, we investigated whether the LC3-binding or dimerization properties of BNIP3/NIX are required for their accumulation. To this end, we generated mCherry-tagged BNIP3/NIX proteins with an LIR motif mutation (LIRm) or dimerization deficiency (OMP25C), in which the TM domains of BNIP3/NIX were replaced with that of OMP25, another OMM protein (Fig. 8 A). Immunoblot analysis confirmed that the WT BNIP3 and NIX proteins, but neither of the OMP25C mutants, formed dimers (Fig. S5, D and E). Using the mt-Keima assay, we found that expression of the WT and OMP25C forms of BNIP3/NIX, but not the LIRm mutants, rescued mitophagy activity in B/N DKO cells (Fig. S5 F). Furthermore, analysis of the immunofluorescence intensities of these proteins revealed strong localization of the WT and OMP25C forms of BNIP3/NIX, but not LIRm, in the mitophagosomes of B/N DKO cells (Fig. 8, B–E). These results suggested that LC3-binding, rather than dimerization, is required for the accumulation of BNIP3/NIX at mitophagosome formation sites.

BNIP3/NIX are known to be phosphorylated at serine residues near the LIR motifs, enhancing their LC3 binding and mitophagy (Fig. 8 F) (Poole et al., 2021; Rogov et al., 2017; Zhu et al., 2013). To investigate the effects of this phosphorylation on BNIP3/NIX assembly, we expressed phospho-mimetic or phospho-dead mutants of BNIP3 or NIX in B/N DKO cells. As reported previously, expression of phospho-mimetic mutants (BNIP3 S17D or NIX S34D/S35D) fully restored mitophagy levels to those of cells expressing the corresponding WT proteins. By contrast, phospho-dead mutants (BNIP3 S17A or NIX S34A/S35A) led to significantly lower levels of mitophagy compared with WT proteins (Fig. S5 G). Consistently, while the phospho-mimetic mutants accumulated in mitophagosomes to the same extent as the WT proteins, the phospho-dead mutant did not, showing a significant decrease compared with WT proteins (Fig. 8, G and H; and Fig. S5, H and I). These results suggested that the interactions between BNIP3/NIX and LC3 are regulated by the phosphorylation of BNIP3/NIX during DFP treatment, supporting the conclusion that the LC3 binding of these receptors promotes their accumulation and mitophagy.

### Distance between the IM and the mitochondrial surface is small in receptor-mediated mitophagy

**Figure 9.**
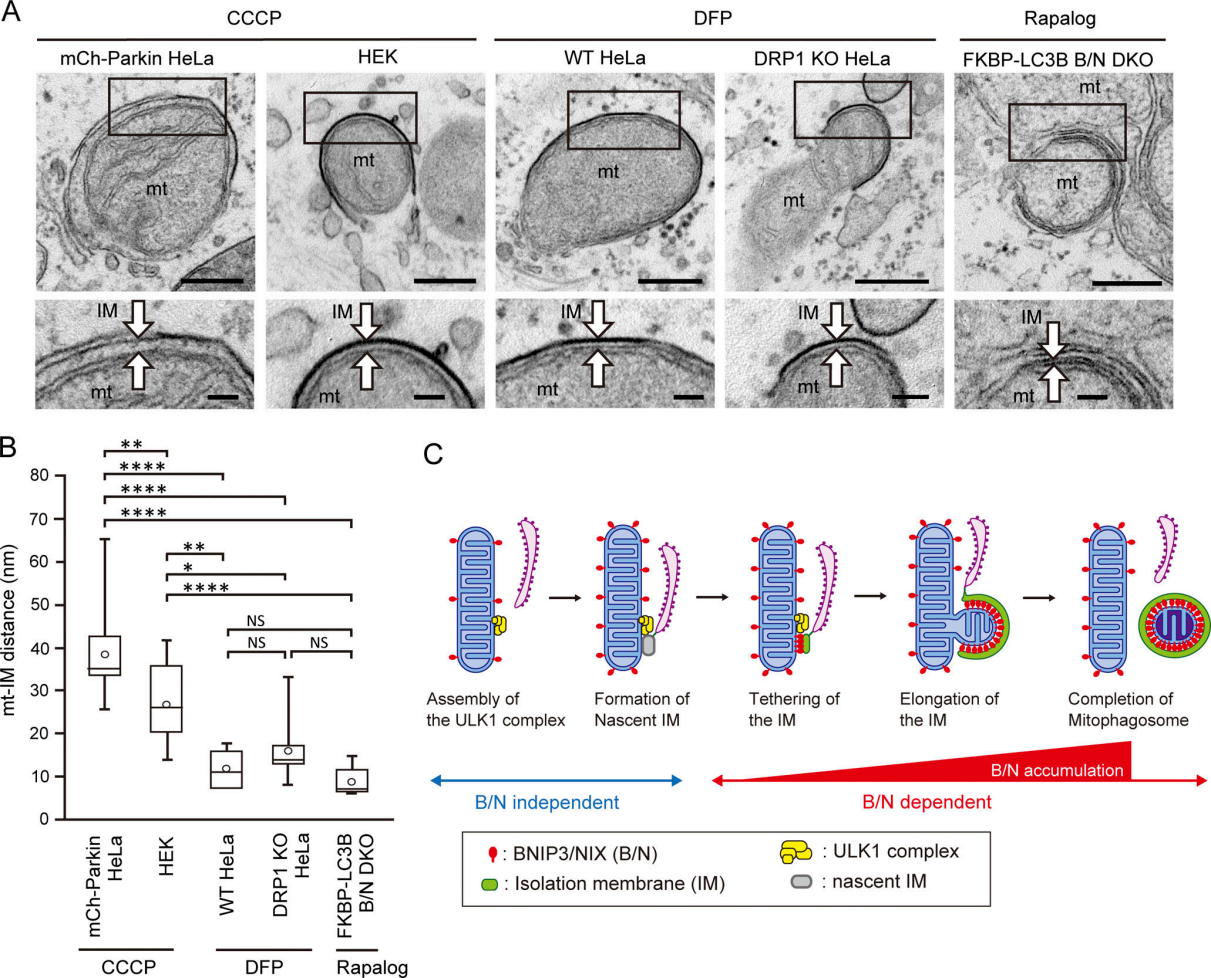
**Tight attachment of the IM to the mitochondrial surface. (A)** EM images of CCCP-treated HeLa cells expressing mCherry-Parkin (mCh-Parkin HeLa) and WT HEK293 cells (HEK), DFP-treated WT and DRP1 KO HeLa cells, and rapalog-treated B/N DKO HeLa cells expressing FKBP-LC3B (FKBP-LC3B B/N DKO). Boxed areas are enlarged and shown below. The arrows indicate OMM and IM. mt, mitochondria. Scale bars: 200 nm (upper row) and 100 nm (lower row). **(B)** Box and whisker plot of the mean distance (open circles) between the OMM and the IM under CCCP-induced mitophagy (mCherry-Parkin HeLa, *n* = 16; HEK, *n* = 11), DFP-induced mitophagy (WT HeLa, *n* = 6; DRP1 KO HeLa, *n* = 10), and rapalog-induced mitophagy (FKBP-LC3B B/N DKO, *n* = 11); *P < 0.05, **P < 0.01, ****P < 0.0001, determined by a Tukey–Kramer test. **(C)** Model of receptor-mediated mitophagy. At the initiation of mitophagy, ULK1 complex and nascent IM are recruited to the mitochondrial surface independently of BNIP3/NIX (B/N). The IM is tethered to the mitochondrial surface, where it elongates and ultimately forms a mitophagosome, depending on B/N. During elongation of the IM, B/N accumulate, promoting tight attachment of the IM to the mitochondria, while ER contacts the IM rim through linear structures.

Considering the tight attachment of the IM to the mitochondrial surface as a characteristic feature of receptor-mediated mitophagy, we measured and compared the distance between IMs and the OMM in receptor-mediated and ubiquitin-mediated mitophagy (Fig. 9, A and B). Ubiquitin-mediated mitophagy, induced by treatment with carbonyl cyanide m-chlorophenyl hydrazone (CCCP), was examined in two cell types: HEK293 cells, which reportedly express moderate levels of endogenous Parkin (Narendra et al., 2008), an E3 ubiquitin ligase; and HeLa cell expressing mCherry-tagged Parkin. The distance between IMs and the OMM (mean ± SD) under CCCP-induced mitophagy was 38.2 ± 10.4 nm in mCherry-Parkin–expressing HeLa cells and 26.7 ± 9.3 nm in HEK293 cells. By contrast, the distances measured in the aforementioned receptor-mediated mitophagy were much smaller, with results in WT (12 ± 4.5 nm) and DRP1 KO (15.9 ± 6.6 nm) HeLa cells. The small distance was comparable with that in WT HeLa cells expressing GFP-ULK1 (13.1 ± 6.1 nm; Fig. 5 B), which were fixed using distinct fixation method for CLEM. These results suggested that the mechanisms enabling attachment of IMs to the mitochondrial surface are different between the two types of mitophagy. Furthermore, the small distance was also comparable with that induced by LC3B-mediated tethering to the mitochondrial surface in B/N DKO HeLa cells (8.7 ± 3.1 nm), suggesting that the receptor–LC3 interaction contributes to the tight association of the IM to mitochondria (Fig. 9 B).

## Discussion

Although live-cell imaging has been used to capture the overall process of DFP/hypoxia-induced mitophagy (Yamashita et al., 2016), the morphological detail at the EM level was limited. Therefore, in this study, we sought to further investigated the morphological features demonstrating that this type of mitophagy is characterized by the tight attachment of the ER-connected IM to a portion of the mitochondrial surface. To elucidate the molecular mechanisms underlying this feature, we focused the role of the mitophagy receptors BNIP3 and NIX, which were recently found to be essential for DFP/hypoxia-induced mitophagy (Yamashita et al., 2024), in the tight attachment of the IM to mitochondria. We discovered that the involvement of these two receptors in the tethering and elongation of IMs takes place after the recruitment of ULK1 complex on the mitochondrial surface (Fig. 9 C). Furthermore, we found that BNIP3/NIX accumulated with LC3 within a limited area on the mitochondrial surface to which the IM was attached (Fig. 9 C). A Similar phenomenon was recently been reported in CoCl_2_-induced mitophagy in U2OS cells, where BNIP3/NIX were found to be co-enriched with TMEM11 and LC3 at mitophagosome formation sites (Gok et al., 2023). The present study recapitulated those findings under different experimental conditions, further demonstrating that the accumulation of BNIP3/NIX was dependent on their LIR motif but not their dimerization property. This finding appears to contradict previous studies demonstrating that homodimerization promotes mitophagy (Hanna et al., 2012; Marinkovic et al., 2021). This discrepancy could reflect differences in the experimental system; specifically, we assayed DFP-induced B/N DKO cells using mt-Keima, while previous studies used transient overexpression of BNIP3/NIX mutants in WT cells induced with CCCP and/or CoCl_2_ and assayed for the co-localization of BNIP3/NIX with LC3 dots. Bunker et al. (2023) also showed that NIX N terminus artificially tethered to the TM domain of monomeric OMM protein Fis1 efficiently induces mitophagy (Bunker et al., 2023). Therefore, although dimerization of BNIP3/NIX can influence mitophagy efficiency, its role may be relatively less important in DFP-induced mitophagy. Nevertheless, the LIR-dependence of BNIP3/NIX accumulation suggests that LC3 on the IM facilitates their accumulation on the mitochondrial surface. Conversely, it is also conceivable that the accumulation of these receptors ensures the tight association of the IM with the mitochondrial surface.

These receptor properties are somewhat distinct from those of mitophagy adapters that mediate ubiquitin-dependent mitophagy, in which optineurin and NDP52 are recruited to mitophagosome formation sites via binding to ubiquitin as well as LC3/γ-aminobutyric acid type A receptor-associated proteins (Padman et al., 2019). The LIR-dependent recruitment of these adapters promotes mitophagy through further recruitment of the ULK1 complex to mitochondria, rather than direct bridging between the IM and mitochondrial surface. In contrast, BNIP3/NIX are not required for recruitment of the ULK1 complex. Our findings support the notion that BNIP3/NIX play a specific role in bridging the IM to the mitochondrial surface. This notion is supported by the smaller IM-to-mitochondria distances observed in BNIP3/NIX-dependent mitophagy (10–18 nm) compared with those in ubiquitin-mediated mitophagy (20–40 nm). This could be explained by the direct interaction of accumulated BNIP3/NIX on mitochondria with LC3 on the IM. By contrast, ubiquitin-mediated mitophagy involves three layers of molecules (OMM proteins, ubiquitin chains, and adapters) that mediate the interaction between the mitochondrial surface and the IM. A recent study also revealed that these adapter molecules, together with ubiquitinated proteins, behave as a sheet-like liquid condensate (Yang et al., 2024), which may be responsible for the wider distance between the IM and mitochondrial surface in ubiquitin-mediated mitophagy.

The yeast ER-phagy receptor Atg40 reportedly assembles in the ER membrane concomitant with autophagosome formation via binding to Atg8, a yeast homolog of LC3 (Mochida et al., 2020). The Atg40 assemblage promotes ER membrane remodeling, which facilitates complete engulfment of the affected ER portion by the autophagosome. Our previous time-lapse imaging study demonstrated the formation of a mitochondrial protrusion from the main mitochondrial body concurrent with elongation of the IM (Yamashita et al., 2016). The EM images in the present study further showed that IMs tightly covered such protrusions. Therefore, it seems reasonable to assume that the LIR-dependent assembly of BNIP3/NIX, as membrane-spanning receptor proteins, promotes tight binding of IMs to target mitochondria, enabling the deformation and segregation of restricted portion of these mitochondria.

In conventional macroautophagy, ER-IM connections have been proposed to function as sites for the delivery of ER-derived membrane lipids to the forming IM in a process involving the activities of the lipid transfer protein Atg2 (Maeda et al., 2019; Osawa et al., 2019; Valverde et al., 2019) and the lipid scramblases Atg9, TMEM41B, and VMP1 (Ghanbarpour et al., 2021; Huang et al., 2021; Li et al., 2021; Maeda et al., 2020; Matoba et al., 2020). Previous evidence has shown the presence of narrow tubular connections between the IM and ER in starvation-induced macroautophagy (Axe et al., 2008; Hamasaki et al., 2013; Hayashi-Nishino et al., 2009; Karanasios et al., 2016; Uemura et al., 2014; Yla-Anttila et al., 2009). More recently, cryo-electron tomography was used to identify stick-like molecular densities at the rim of the IM within ER-IM contact sites in aggrephagy (Carter et al., 2020) and xenophagy (Li et al., 2023). In the present study, IM-ER contact sites were frequently observed on the rim of the IM, forming short linear structures at the early phase of IM expansion and transforming into tubular and linear structures at the late phase. The tubular and linear structures observed at the late phase (Fig. 2) may correspond to the structures referred to as IM-associated tubules in a previous study (Uemura et al., 2014). Considering that lipid supply systems might cease at a final closing phase, the short linear structures observed at the early phase (Fig. 3) could represent functional ER-IM contact sites. It has yet to be elucidated whether these short linear structures correspond to the stick-like molecular densities observed under cryo-electron tomography. Nevertheless, these observations strongly suggest that IM growth in receptor-mediated mitophagy is supported by the ER-derived lipid supply at the rim of the IM (Fig. 9 C).

In BNIP3/NIX-DKO cells, ULK1 and LC3 are recruited to mitochondria, with ER and IM-like structures occasionally found attached to mitochondria. These structures are most likely nascent or precursor forms of IMs called omegasomes. Therefore, the initial contact of a nascent IM to a mitochondrion appears to be independent of the LC3–BNIP3/NIX interaction. Recent studies demonstrated that the BNIP3/NIX–WIPIs interactions could be important for robust mitophagy induction (Adriaenssens et al., 2024, *Preprint*; Bunker et al., 2023). Consistently, we found that BNIP3/NIX interacted with WIPI2 during DFP treatment (Fig. S4 D). However, LC3 and nascent IM were recruited to the mitochondrial surface in the absence of BNIP3/NIX proteins (Figs. 4 and 5), suggesting that the BNIP3/NIX–WIPI2 interactions are not crucial for this initial event. Collectively, these findings support the idea that the BNIP3/NIX–WIPI2 interactions at the rim of the IM is involved in its precise expansion along the mitochondrial surface. Further study is needed to clarify the molecular nature of nascent IMs.

In conclusion, our data support a new model of receptor-mediated mitophagy. In contrast to the alternative model, which suggests that a large, preformed IM near ER engulfs a portion of the target mitochondrion, we propose that the autophagy initiation machinery recognizes a specific region of the mitochondrion as the site for mitophagosome formation, initiating IM formation. In this process, BNIP3/NIX ensure that the IM is tethered to the mitochondrial surface. As the tethered IM expands, the local accumulation of BNIP3/NIX in association with LC3 promotes a tight attachment between the IM and the mitochondrial surface, facilitating the complete engulfment of the targeted mitochondrial region to form a mitophagosome (Fig. 9 C).

## Materials and methods

### Cell lines, cell culture, and induction of mitophagy

All cell lines used in this study and their sources are listed in Table 1. WT, DRP1 KO HeLa, and B/N DKO cells co-expressing GFP-LC3B and mito-mCherry, and WT, B/N DKO, and FIP200 KO HeLa cells expressing mt-Keima were generated as previously described (Yamashita et al., 2016, 2024). HeLa Kyoto cells stably expressing mCherry-Parkin were generated as previously described (Teranishi et al., 2022). All HeLa cells and HEK293 cells were maintained in high-glucose DMEM supplemented with 10% FBS, 100 U/ml penicillin, and 100 μg/ml streptomycin (hereafter referred to as control medium) at 37°C under 5% CO_2_. For DFP-induced mitophagy, cells were cultured in control medium containing 1 mM DFP (#379409; Sigma-Aldrich) for 16 or 24 h. For Parkin-dependent mitophagy, mCherry-Parkin–expressing HeLa Kyoto cells and HEK293 cells were cultured in control medium containing 10 μM CCCP (#C2759; Sigma-Aldrich) for 9 and 4 h, respectively.

**Table 1.** Cell lines used in this study

Cell line	Source
HeLa (WT for Drp1 KO, FIP200 KO, and B/N DKO HeLa cells)	Yamashita et al. (2016)
Drp1 KO HeLa	Yamashita et al. (2016)
FIP200 KO HeLa	Yamashita et al. (2016)
BNIP3/NIX DKO HeLa	Yamashita et al. (2024)
HeLa Kyoto cells stably expressing mCherry-Parkin	Tamotsu Yoshimori’s lab. (Osaka University, Osaka, Japan)
HEK293	N/A
HEK293T	N/A

### Plasmids

All plasmids used in this study and their sources are listed in Table 2. To construct a GFP-tagged ULK1 vector, sequences encoding EGFP and human ULK1 were amplified by PCR and cloned into the PacI-BamHI and BamHI-EcoRI sites of pMXs-puro (RTV-012; Cell Biolabs), respectively. To construct mCherry-tagged WT and LIRm forms of BNIP3/NIX, sequences encoding mCherry and BNIP3/NIX were amplified by PCR and cloned into the PacI-BamHI and BamHI-EcoRI sites, respectively, of the pQCXIB (W297-1) (gift from Eric Campeau, University of Massachusetts Medical School, Worcester, MA, USA [RRID:Addgene_22800]). To construct the OMP25C forms, the TM domains of mCherry-BNIP3 (164–194 aa in BNIP3) and mCherry-NIX (188–219 aa in NIX) were replaced with the C-terminal sequence of human OMP25 (101–145 aa; OMP25C) using the In-Fusion method (#638947; Clontech). The WT, LIRm, and OMP25C forms of mCherry-BNIP3/NIX were then transferred to pQCXIH (gift from Joseph Nevins, Duke University School of Medicine, Durham, NC, USA [RRID:Addgene_37106]) using the PacI-EcoRI sites. Before employing pQCXIH, the EcoRI site in the hygromycin-resistance gene was eliminated using inverse PCR-based mutagenesis, resulting in pQCXIHm. To construct FRB-GFP-OMP25C, the FRB region of mTOR1 (2021–2113 aa) and OMP25C were amplified by PCR and cloned into the PacI-BamHI and BamHI-EcoRI sites of pQCXIB (W297-1), respectively. The PCR-amplified coding sequence of EGFP was then inserted between FRB and OMP25C using the In-Fusion method. The T2098L mutation was introduced in FRB using inverse PCR-based mutagenesis. To construct FKBP-tagged ULK1 and LC3B, the coding sequences of FKBP1A, ULK1, and LC3B were amplified by PCR, and then FKBP and ULK1/LC3B were cloned into the PacI-BamHI and BamHI-EcoRI sites in pQCXIHm, respectively. To construct mCherry-tagged Parkin, sequences encoding Parkin and mCherry were amplified by PCR and cloned into pMRXIP.

**Table 2.** Plasmids used in this study

Plasmid	Source
pMRXIP-GFP-LC3B	Yamashita et al. (2016)
pMXs-Neo-mt-mCherry	Yamashita et al. (2016)
pMXs-Puro	Cell Biolabs Inc. (RTV-012)
pQCXIB	Addgene (#22800)
pQCXIH	Addgene (#37106)
pQCXIHm	This study
pMXs-Puro-GFP-ULK1	This study
pQCXIB-BNIP3_WT	Yamashita et al. (2024)
pQCXIB-BNIP3_LIRm	Yamashita et al. (2024)
pQCXIB-BNIP3-OMP25C	This study
pQCXIB-BNIP3_S17D	This study
pQCXIB-BNIP3_S17A	This study
pQCXIB-NIX_WT	Yamashita et al. (2024)
pQCXIB-NIX_LIRm	Yamashita et al. (2024)
pQCXIB-NIX-OMP25C	This study
pQCXIB-NIX_S34D/S35D	This study
pQCXIB-NIX_S34A/S35A	This study
pQCXIHm-mCherry-BNIP3_WT	This study
pQCXIHm-mCherry-BNIP3_LIRm	This study
pQCXIHm-mCherry-BNIP3-OMP25C	This study
pQCXIHm-mCherry-BNIP3_S17D	This study
pQCXIHm-mCherry-BNIP3_S17A	This study
pQCXIHm-mCherry-NIX_WT	This study
pQCXIHm-mCherry-NIX_LIRm	This study
pQCXIHm-mCherry-NIX-OMP25C	This study
pQCXIHm-mCherry-NIX_S34D/S35D	This study
pQCXIHm-mCherry-NIX_S34A/S35A	This study
pQCXIB-FRB-GFP-OMP25C	This study
pQCXIHm-FKBP alone	This study
pQCXIHm-FKBP-ULK1	This study
pQCXIHm-FKBP-LC3B	This study
pMRXIP-mCherry-Parkin	This study

### Retroviral transduction

For stable expression of exogenous genes, retroviral transduction of cells was performed as follows. HEK293T cells were co-transfected with pUMVC (RRID:Addgene_8449), pVSV-G (RRID:Addgene_8454), and the retroviral vector using polyethyleneimine MAX (#24765; Polysciences). At 16 h after transfection, the medium was replaced with fresh medium, and the cells were cultured for an additional 24 h. Retroviral supernatants were then collected and filtered. HeLa cells were transduced with retroviral vectors in the presence of 8 μg/ml polybrene (H9268; Sigma-Aldrich). At 24 h after transduction, the retroviral supernatant was replaced with fresh medium containing an antibiotic (25 μg/ml blasticidin, 300 μg/ml hygromycin, or 1 μg/ml puromycin).

### Antibodies

The following primary antibodies were used in this study: rabbit polyclonal anti-Tom20 (sc-11415; RRID:AB_2207533; Santa Cruz Biotechnology), anti-RFP (#PM005, RRID:AB_591279; MBL), anti-LC3 (PM036, RRID:AB_2274121; MBL), anti-FUNDC1 (ARP53280_P050, RRID:AB_1294254; Aviva Systems Biology), anti-BCL2L13 (16612-1-AP, RRID:AB_1850928; Proteintech) antibodies, rabbit monoclonal anti-BNIP3 (44060; RRID:AB_2799259; Cell Signaling Technology), anti-NIX (12396; RRID:AB_2688036; Cell Signaling Technology), anti-Atg13 (13468; RRID:AB_2797419; Cell Signaling Technology) antibodies, mouse monoclonal anti-Tom20 (sc-17764, RRID:AB_628381; Santa Cruz Biotechnology), anti-LC3 (CTC-LC3-2-IC; RRID:AB_10707197; CosmoBio), anti-Atg14 (M184-3, RRID:AB_10897331; MBL), anti-WIPI2 (ab105459, RRID:AB_10860881; Abcam), and anti-FKBP8 (MAB3580, RRID:AB_2262675; R&D systems) antibodies. Alexa Fluor 647–conjugated secondary antibody against mouse IgG (RRID:AB_2535804) and Alexa Fluor 488– (RRID:AB_2534114) or 594– (RRID:AB_2534116) conjugated secondary antibody against rabbit IgG were purchased from Thermo Fisher Scientific.

### Immunofluorescence microscopy

Cells were fixed with 4% PFA in PBS at room temperature for 15 min, permeabilized with 0.1% digitonin in PBS for 10 min, and blocked with 0.4% BSA in PBS for 10 min. The cells were then incubated with primary antibodies for 1 h, followed by fluorescently labeled secondary antibodies for 30 min in the blocking buffer. Fluorescence images were obtained using a confocal microscope: FV1000 or FV1200 (Olympus) equipped with a PlanApo N lens (60×/NA 1.42) and a UPlan SApo lens (100×/NA 1.40) a ZEISS LSM700 equipped with a Plan-Apochromat lens (63×/NA 1.4).

### Live-cell imaging

HeLa cells expressing GFP-LC3B and mCherry-BNIP3/NIX were cultured in an 8-well chambered coverglass (#155409; Thermo Fisher Scientific) overnight at 37°C under 5% CO_2_. The cells were stained with 100 nM MitoTracker Deep Red (#M22426; Thermo Fisher Scientific) for 15 min and then washed with control medium. Following the MitoTracker staining, control medium was replaced with DFP-containing medium, and the cells were cultured for an additional 12 h. The chambered coverglass was transferred to the stage-top incubator for 1 h at 37°C under 5% CO_2_, after which time-lapse analysis was started using a Zeiss LSM710 confocal microscope with a Plan-Apochromat 63×/1.4 oil objective lens. Time-lapse images were acquired at 20-s intervals and processed by Fiji ImageJ (1.54f, Schindelin et al., 2012, RRID:SCR_003070).

### CLEM analysis

Procedures for CLEM with reduced osmium fixation were previously described (Arai and Waguri, 2019). Briefly, HeLa cells were cultured on coverslips with a 150-μm grid and position labels (#GC1310; Matsunami Glass Industry), fixed with 2% PFA–0.1 or 0.5% glutaraldehyde (GA) in 0.1 M phosphate buffer (PB; pH 7.4) for 15 min at room temperature, and rinsed with 0.1 M PB (pH 7.4). To visualize mitochondria, MitoTracker Deep Red stock solution was added to the culture medium to a final concentration of 100 nM. After incubation for 15 min, the cells were rinsed with control medium once and immediately fixed with the same fixative, as described above. Fluorescence images were acquired at specific position numbers using a confocal microscope FV1000, as described above. The cells were fixed again with 2% PFA–2% GA in 0.1 M PB (pH 7.4) for 15 min and stored at 4°C. They were then postfixed with 1% OsO_4_–1.5% tetrapotassium ferrocyanide in 0.1 M PB (pH 7.4) for 60 min at room temperature. After dehydration and resin embedding, areas containing cells of interest were trimmed from the resin based on the fluorescence images, and serial ultrathin (60-nm thickness) sections were prepared and observed with a transmission EM (JEM-1400; JEOL). Fluorescence and EM images were positionally aligned based on the distribution and morphology of mitochondria in cells, using Photoshop CS6 software (RRID:SCR_014199; Adobe).

### Aldehyde-osmium simultaneous fixation and electron tomography

Procedures for aldehyde-osmium fixation and electron tomography were previously described (Arai and Waguri, 2019). Briefly, cells were fixed with 2% PFA–2% GA–2% OsO_4_ for 60 min at room temperature. Resin embedding and EM observation of the serial ultrathin sections were carried out as described above. For electron tomography, 300-nm–thick sections were prepared and mounted on thin bar grids (#G200HH Cu; Gilder). Tilt image series were captured at a magnification of 10,000× (Fig. 2) or 8,000× (Fig. 3) using a JEM-1400 electron microscope at 120 kV. Images were taken at 1-degree intervals over a tilt range of ±60° using a charge-coupled device camera. The recording and subsequent 3D reconstruction were carried out using TEMography software (System In Frontier) and the 3DMOD program included in the IMOD software package (Kremer et al., 1996, RRID:SCR_003297). A 3D model was produced by Amira software (FEI, RRID:SCR_007353).

### Measurements of distance between IMs and mitochondria

To measure the average distance between the mitochondrial surface and the IM, the area of the gap was measured using Fiji ImageJ and then divided by the length of the OMM covered by the IM. In the case of an IM that was located near a mitochondrion but had not expanded along its surface or was engulfing a portion of cytoplasm, the shortest distance was measured.

### CLEM/FIB-scanning EM analysis

CLEM was performed as described above. The trimmed resin block containing the area of interest was mounted onto scanning EM pins. Samples were analyzed using a FEI Helios Nanolab 650 DualBeam FIB-scanning EM system. The block was sequentially cut and imaged using Auto Slice and View G3 V1.3 software. The slice thickness was set to 10 nm. The resulting stack of images was aligned and reconstructed using Fiji ImageJ (RRID: SCR_003070) and Amira software (RRID:SCR_007353).

### Immunoprecipitation

Cells were cultured with 1 mM DFP in 10 cm for 12 h and then treated with 100 nM bafilomycin A1 for additional 12 h with DFP. The cells were lysed with 0.5% triton X-100–containing buffer, and immunoprecipitation was performed by using RFP-trap magnetic agarose (rtma, RRID:AB_2631363; Proteintech), according to the manufacturer’s instruction. IP products were subjected to immunoblot analysis. After SDS-PAGE, proteins were transferred to PVDF membranes by semidry blotting. The membranes were blocked with PBS-T containing 5% skim milk at room temperature for 30 min, then incubated with primary antibodies in PBS-T containing 2% skim milk at 4°C overnight. After incubation with primary antibodies, the membranes were washed with PBS-T and then incubated with HRP-conjugated secondary antibodies diluted in the same buffer as the primary antibodies at room temperature for 2 h. After incubation with secondary antibodies, the membranes were washed with PBS-T. The membranes were incubated with HRP substrate EzWestLumi plus (WSE-7120L; ATTO), and then the signals were captured using ChemiDoc XRS^+^ (Bio-Rad) or Touch Imager (e-BLOT).

### Mitophagy assay

Mitophagy assays were conducted using a pH-sensitive fluorescent protein, Keima containing a mitochondrial-targeting sequence fused to its N terminus (mt-Keima). The excitation peak of mt-Keima shifts from 440 to 586 nm in an environmental pH-dependent manner, thereby enabling the distinct identification of mitochondria (neutral pH) and mitolysosomes (acidic pH), which are excited by 440 and 586 nm light, respectively, during mitophagy (Katayama et al., 2011; Yamashita and Kanki, 2018). Mitophagy assays were performed using imaging cytometry, employing an ImageXpress Micro XLS system (RRID:SCR_025259; Molecular Devices) with a Plan Apo 60× objective lens (Nikon). HeLa cells expressing mt-Keima were seeded in a 96-well glass-bottom plate at a density of 2 × 10^4^ cells per well and cultured overnight. Unless otherwise noted, mitophagy was induced by chemical treatment for 24 h, after which 2 μg/ml Hoechst33342 (H342; DOJINDO) was added to each well. The plate was subjected to imaging cytometry with fluorescence filters for Texas Red and DAPI to detect mitophagosomes and nuclei, respectively. Imaging cytometry data were analyzed by MetaXpress software (RRID: SCR_016654; Molecular Devices).

### Statistical analyses

Quantification of signal number and intensity in fluorescence microscopy was analyzed using one-way ANOVA followed by the Tukey–Kramer or the Dunnett’s post hoc test, the Kruskal–Wallis test followed by the Steel–Dwass post hoc test or the Mann–Whitney U test, as described in the figure legends. Distance between mitochondria and IMs in EM were analyzed using either the Mann–Whitney U test or the Tukey–Kramer test, as indicated in the figure legends. For the Tukey–Kramer test, data distribution was assumed to be normal, but this was not formally tested.

### Online supplemental material

Fig. S1 shows the morphology of receptor-mediated mitophagy revealed by CLEM (related to Fig. 1 A). Fig. S2 shows the analysis of the fine morphology of receptor-mediated mitophagy. Fig. S3 shows the mitolysosome formation and recruitment of ULK1 and IM in BNIP3/NIX DKO cells. Fig. S4 shows the effects of artificial tethering of the FKBP domain, interaction analysis of BNIP3/NIX with other autophagy factors, and serial ultrathin sections related to Fig. 7 G. Fig. S5 shows that the BNIP3/NIX are accumulated in mitophagosome in an LIR-dependent manner (related to Fig. 8). Video1 shows overall morphology of receptor-mediated mitophagy revealed by CLEM-FIB–scanning EM (related to Fig. 1, C and D). Video 2 shows the distribution of IM-ER contact sites on the IM (related to Fig. 1 E). Video 3 shows the overall morphology of receptor-mediated mitophagy in the later phase revealed by electron tomography (related to Fig. 2, A–C). Video 4 shows the linear structures connecting ER with the IM (related to Fig. 2, D and E). Video 5 shows the linear/tubular structures connecting ER with the IM (related to Fig. 2, F and G). Video 6 shows the overall morphology of receptor-mediated mitophagy in the early phase, as revealed by electron tomography (related to Fig. 3, A–D). Video 7 shows the linear structures connecting ER with the IM (related to Fig. 3, E, F, and H). Video 8 shows the linear structures connecting ER with the IM (related to Fig. 3, G and I).

## Supplementary Material

SourceData FS3 is the source file for Fig. S3.

SourceData FS4 is the source file for Fig. S4.

SourceData FS5 is the source file for Fig. S5.

## Data Availability

The data underlying this study are available from the corresponding authors (TK or SW) upon reasonable request.

## References

[bib1] Adriaenssens, E., S. Schaar, A.S.I. Cook, J.F.M. Stuke, J. Sawa-Makarska, T.N. Nguyen, X. Ren, M. Schuschnig, J. Romanov, G. Khuu, . 2024. Reconstitution of BNIP3/NIX-mediated autophagy reveals two pathways and hierarchical flexibility of the initiation machinery. bioRxiv. 10.1101/2024.08.28.609967 (Preprint posted August 28, 2024). PMC12339401 40715440

[bib2] Arai, R., and S. Waguri. 2019. Improved electron microscopy fixation methods for tracking autophagy-associated membranes in cultured mammalian cells. Methods Mol. Biol. 1880:211–221. 10.1007/978-1-4939-8873-0_13 30610699

[bib3] Axe, E.L., S.A. Walker, M. Manifava, P. Chandra, H.L. Roderick, A. Habermann, G. Griffiths, and N.T. Ktistakis. 2008. Autophagosome formation from membrane compartments enriched in phosphatidylinositol 3-phosphate and dynamically connected to the endoplasmic reticulum. J. Cell Biol. 182:685–701. 10.1083/jcb.200803137 18725538 PMC2518708

[bib4] Bhujabal, Z., A.B. Birgisdottir, E. Sjøttem, H.B. Brenne, A. Øvervatn, S. Habisov, V. Kirkin, T. Lamark, and T. Johansen. 2017. FKBP8 recruits LC3A to mediate Parkin-independent mitophagy. EMBO Rep. 18:947–961. 10.15252/embr.201643147 28381481 PMC5452039

[bib5] Bunker, E.N., F. Le Guerroué, C. Wang, M.P. Strub, A. Werner, N. Tjandra, and R.J. Youle. 2023. Nix interacts with WIPI2 to induce mitophagy. EMBO J. 42:e113491. 10.15252/embj.2023113491 37621214 PMC10646555

[bib6] Carter, S.D., J.I. Mamede, T.J. Hope, and G.J. Jensen. 2020. Correlated cryogenic fluorescence microscopy and electron cryo-tomography shows that exogenous TRIM5α can form hexagonal lattices or autophagy aggregates in vivo. Proc. Natl. Acad. Sci. USA. 117:29702–29711. 10.1073/pnas.1920323117 33154161 PMC7703684

[bib7] Ganley, I.G., and A. Simonsen. 2022. Diversity of mitophagy pathways at a glance. J. Cell Sci. 135:jcs259748. 10.1242/jcs.259748 36504076 PMC10656428

[bib8] Ghanbarpour, A., D.P. Valverde, T.J. Melia, and K.M. Reinisch. 2021. A model for a partnership of lipid transfer proteins and scramblases in membrane expansion and organelle biogenesis. Proc. Natl. Acad. Sci. USA. 118:e2101562118. 10.1073/pnas.2101562118 33850023 PMC8072408

[bib9] Gok, M.O., O.M. Connor, X. Wang, C.J. Menezes, C.B. Llamas, P. Mishra, and J.R. Friedman. 2023. The outer mitochondrial membrane protein TMEM11 demarcates spatially restricted BNIP3/BNIP3L-mediated mitophagy. J. Cell Biol. 222:e202204021. 10.1083/jcb.202204021 36795401 PMC9960330

[bib10] Hamasaki, M., N. Furuta, A. Matsuda, A. Nezu, A. Yamamoto, N. Fujita, H. Oomori, T. Noda, T. Haraguchi, Y. Hiraoka, . 2013. Autophagosomes form at ER-mitochondria contact sites. Nature. 495:389–393. 10.1038/nature11910 23455425

[bib11] Hanna, R.A., M.N. Quinsay, A.M. Orogo, K. Giang, S. Rikka, and A.B. Gustafsson. 2012. Microtubule-associated protein 1 light chain 3 (LC3) interacts with Bnip3 protein to selectively remove endoplasmic reticulum and mitochondria via autophagy. J. Biol. Chem. 287:19094–19104. 10.1074/jbc.M111.322933 22505714 PMC3365942

[bib12] Hayashi-Nishino, M., N. Fujita, T. Noda, A. Yamaguchi, T. Yoshimori, and A. Yamamoto. 2009. A subdomain of the endoplasmic reticulum forms a cradle for autophagosome formation. Nat. Cell Biol. 11:1433–1437. 10.1038/ncb1991 19898463

[bib13] Heo, J.M., A. Ordureau, J.A. Paulo, J. Rinehart, and J.W. Harper. 2015. The PINK1-PARKIN mitochondrial ubiquitylation pathway drives a program of OPTN/NDP52 recruitment and TBK1 activation to promote mitophagy. Mol. Cell. 60:7–20. 10.1016/j.molcel.2015.08.016 26365381 PMC4592482

[bib14] Huang, D., B. Xu, L. Liu, L. Wu, Y. Zhu, A. Ghanbarpour, Y. Wang, F.J. Chen, J. Lyu, Y. Hu, . 2021. TMEM41B acts as an ER scramblase required for lipoprotein biogenesis and lipid homeostasis. Cell Metab. 33:1655–1670.e8. 10.1016/j.cmet.2021.05.006 34015269

[bib15] Itakura, E., C. Kishi-Itakura, I. Koyama-Honda, and N. Mizushima. 2012. Structures containing Atg9A and the ULK1 complex independently target depolarized mitochondria at initial stages of Parkin-mediated mitophagy. J. Cell Sci. 125:1488–1499. 10.1242/jcs.094110 22275429

[bib16] Karanasios, E., S.A. Walker, H. Okkenhaug, M. Manifava, E. Hummel, H. Zimmermann, Q. Ahmed, M.C. Domart, L. Collinson, and N.T. Ktistakis. 2016. Autophagy initiation by ULK complex assembly on ER tubulovesicular regions marked by ATG9 vesicles. Nat. Commun. 7:12420. 10.1038/ncomms12420 27510922 PMC4987534

[bib17] Katayama, H., T. Kogure, N. Mizushima, T. Yoshimori, and A. Miyawaki. 2011. A sensitive and quantitative technique for detecting autophagic events based on lysosomal delivery. Chem. Biol. 18:1042–1052. 10.1016/j.chembiol.2011.05.013 21867919

[bib18] Kremer, J.R., D.N. Mastronarde, and J.R. McIntosh. 1996. Computer visualization of three-dimensional image data using IMOD. J. Struct. Biol. 116:71–76. 10.1006/jsbi.1996.0013 8742726

[bib19] Ktistakis, N.T. 2020. ER platforms mediating autophagosome generation. Biochim. Biophys. Acta Mol. Cell Biol. Lipids. 1865:158433. 10.1016/j.bbalip.2019.03.005 30890442

[bib20] Lazarou, M., D.A. Sliter, L.A. Kane, S.A. Sarraf, C. Wang, J.L. Burman, D.P. Sideris, A.I. Fogel, and R.J. Youle. 2015. The ubiquitin kinase PINK1 recruits autophagy receptors to induce mitophagy. Nature. 524:309–314. 10.1038/nature14893 26266977 PMC5018156

[bib21] Li, M., I. Tripathi-Giesgen, B.A. Schulman, W. Baumeister, and F. Wilfling. 2023. In situ snapshots along a mammalian selective autophagy pathway. Proc. Natl. Acad. Sci. USA. 120:e2221712120. 10.1073/pnas.2221712120 36917659 PMC10041112

[bib22] Li, Y.E., Y. Wang, X. Du, T. Zhang, H.Y. Mak, S.E. Hancock, H. McEwen, E. Pandzic, R.M. Whan, Y.C. Aw, . 2021. TMEM41B and VMP1 are scramblases and regulate the distribution of cholesterol and phosphatidylserine. J. Cell Biol. 220:e202103105. 10.1083/jcb.202103105 33929485 PMC8077175

[bib23] Liu, L., D. Feng, G. Chen, M. Chen, Q. Zheng, P. Song, Q. Ma, C. Zhu, R. Wang, W. Qi, . 2012. Mitochondrial outer-membrane protein FUNDC1 mediates hypoxia-induced mitophagy in mammalian cells. Nat. Cell Biol. 14:177–185. 10.1038/ncb2422 22267086

[bib24] Maeda, S., C. Otomo, and T. Otomo. 2019. The autophagic membrane tether ATG2A transfers lipids between membranes. Elife. 8:e45777. 10.7554/eLife.45777 31271352 PMC6625793

[bib25] Maeda, S., H. Yamamoto, L.N. Kinch, C.M. Garza, S. Takahashi, C. Otomo, N.V. Grishin, S. Forli, N. Mizushima, and T. Otomo. 2020. Structure, lipid scrambling activity and role in autophagosome formation of ATG9A. Nat. Struct. Mol. Biol. 27:1194–1201. 10.1038/s41594-020-00520-2 33106659 PMC7718406

[bib26] Marinković, M., M. Šprung, and I. Novak. 2021. Dimerization of mitophagy receptor BNIP3L/NIX is essential for recruitment of autophagic machinery. Autophagy. 17:1232–1243. 10.1080/15548627.2020.1755120 32286918 PMC8143235

[bib27] Matoba, K., T. Kotani, A. Tsutsumi, T. Tsuji, T. Mori, D. Noshiro, Y. Sugita, N. Nomura, S. Iwata, Y. Ohsumi, . 2020. Atg9 is a lipid scramblase that mediates autophagosomal membrane expansion. Nat. Struct. Mol. Biol. 27:1185–1193. 10.1038/s41594-020-00518-w 33106658

[bib28] Mochida, K., A. Yamasaki, K. Matoba, H. Kirisako, N.N. Noda, and H. Nakatogawa. 2020. Super-assembly of ER-phagy receptor Atg40 induces local ER remodeling at contacts with forming autophagosomal membranes. Nat. Commun. 11:3306. 10.1038/s41467-020-17163-y 32620754 PMC7335187

[bib29] Montava-Garriga, L., and I.G. Ganley. 2020. Outstanding questions in mitophagy: What we do and do not know. J. Mol. Biol. 432:206–230. 10.1016/j.jmb.2019.06.032 31299243

[bib30] Murakawa, T., O. Yamaguchi, A. Hashimoto, S. Hikoso, T. Takeda, T. Oka, H. Yasui, H. Ueda, Y. Akazawa, H. Nakayama, . 2015. Bcl-2-like protein 13 is a mammalian Atg32 homologue that mediates mitophagy and mitochondrial fragmentation. Nat. Commun. 6:7527. 10.1038/ncomms8527 26146385 PMC4501433

[bib31] Narendra, D., A. Tanaka, D.F. Suen, and R.J. Youle. 2008. Parkin is recruited selectively to impaired mitochondria and promotes their autophagy. J. Cell Biol. 183:795–803. 10.1083/jcb.200809125 19029340 PMC2592826

[bib32] Novak, I., V. Kirkin, D.G. McEwan, J. Zhang, P. Wild, A. Rozenknop, V. Rogov, F. Löhr, D. Popovic, A. Occhipinti, . 2010. Nix is a selective autophagy receptor for mitochondrial clearance. EMBO Rep. 11:45–51. 10.1038/embor.2009.256 20010802 PMC2816619

[bib33] Osawa, T., T. Kotani, T. Kawaoka, E. Hirata, K. Suzuki, H. Nakatogawa, Y. Ohsumi, and N.N. Noda. 2019. Atg2 mediates direct lipid transfer between membranes for autophagosome formation. Nat. Struct. Mol. Biol. 26:281–288. 10.1038/s41594-019-0203-4 30911189

[bib34] Padman, B.S., T.N. Nguyen, L. Uoselis, M. Skulsuppaisarn, L.K. Nguyen, and M. Lazarou. 2019. LC3/GABARAPs drive ubiquitin-independent recruitment of Optineurin and NDP52 to amplify mitophagy. Nat. Commun. 10:408. 10.1038/s41467-019-08335-6 30679426 PMC6345886

[bib35] Pickles, S., P. Vigié, and R.J. Youle. 2018. Mitophagy and quality control mechanisms in mitochondrial maintenance. Curr. Biol. 28:R170–R185. 10.1016/j.cub.2018.01.004 29462587 PMC7255410

[bib36] Poole, L.P., A. Bock-Hughes, D.E. Berardi, and K.F. Macleod. 2021. ULK1 promotes mitophagy via phosphorylation and stabilization of BNIP3. Sci. Rep. 11:20526. 10.1038/s41598-021-00170-4 34654847 PMC8519931

[bib37] Putyrski, M., and C. Schultz. 2012. Protein translocation as a tool: The current rapamycin story. FEBS Lett. 586:2097–2105. 10.1016/j.febslet.2012.04.061 22584056

[bib38] Rambold, A.S., B. Kostelecky, N. Elia, and J. Lippincott-Schwartz. 2011. Tubular network formation protects mitochondria from autophagosomal degradation during nutrient starvation. Proc. Natl. Acad. Sci. USA. 108:10190–10195. 10.1073/pnas.1107402108 21646527 PMC3121813

[bib39] Richter, B., D.A. Sliter, L. Herhaus, A. Stolz, C. Wang, P. Beli, G. Zaffagnini, P. Wild, S. Martens, S.A. Wagner, . 2016. Phosphorylation of OPTN by TBK1 enhances its binding to Ub chains and promotes selective autophagy of damaged mitochondria. Proc. Natl. Acad. Sci. USA. 113:4039–4044. 10.1073/pnas.1523926113 27035970 PMC4839414

[bib40] Rogov, V.V., H. Suzuki, M. Marinković, V. Lang, R. Kato, M. Kawasaki, M. Buljubašić, M. Šprung, N. Rogova, S. Wakatsuki, . 2017. Phosphorylation of the mitochondrial autophagy receptor Nix enhances its interaction with LC3 proteins. Sci. Rep. 7:1131. 10.1038/s41598-017-01258-6 28442745 PMC5430633

[bib41] Schindelin, J., I. Arganda-Carreras, E. Frise, V. Kaynig, M. Longair, T. Pietzsch, S. Preibisch, C. Rueden, S. Saalfeld, B. Schmid, . 2012. Fiji: An open-source platform for biological-image analysis. Nat. Methods. 9:676–682. 10.1038/nmeth.2019 22743772 PMC3855844

[bib42] Schweers, R.L., J. Zhang, M.S. Randall, M.R. Loyd, W. Li, F.C. Dorsey, M. Kundu, J.T. Opferman, J.L. Cleveland, J.L. Miller, and P.A. Ney. 2007. NIX is required for programmed mitochondrial clearance during reticulocyte maturation. Proc. Natl. Acad. Sci. USA. 104:19500–19505. 10.1073/pnas.0708818104 18048346 PMC2148318

[bib43] Tanaka, A., M.M. Cleland, S. Xu, D.P. Narendra, D.F. Suen, M. Karbowski, and R.J. Youle. 2010. Proteasome and p97 mediate mitophagy and degradation of mitofusins induced by Parkin. J. Cell Biol. 191:1367–1380. 10.1083/jcb.201007013 21173115 PMC3010068

[bib44] Teranishi, H., K. Tabata, M. Saeki, T. Umemoto, T. Hatta, T. Otomo, K. Yamamoto, T. Natsume, T. Yoshimori, and M. Hamasaki. 2022. Identification of CUL4A-DDB1-WDFY1 as an E3 ubiquitin ligase complex involved in initiation of lysophagy. Cell Rep. 40:111349. 10.1016/j.celrep.2022.111349 36103833

[bib45] Twig, G., A. Elorza, A.J. Molina, H. Mohamed, J.D. Wikstrom, G. Walzer, L. Stiles, S.E. Haigh, S. Katz, G. Las, . 2008. Fission and selective fusion govern mitochondrial segregation and elimination by autophagy. EMBO J. 27:433–446. 10.1038/sj.emboj.7601963 18200046 PMC2234339

[bib46] Uemura, T., M. Yamamoto, A. Kametaka, Y.S. Sou, A. Yabashi, A. Yamada, H. Annoh, S. Kametaka, M. Komatsu, and S. Waguri. 2014. A cluster of thin tubular structures mediates transformation of the endoplasmic reticulum to autophagic isolation membrane. Mol. Cell. Biol. 34:1695–1706. 10.1128/MCB.01327-13 24591649 PMC3993601

[bib47] Valverde, D.P., S. Yu, V. Boggavarapu, N. Kumar, J.A. Lees, T. Walz, K.M. Reinisch, and T.J. Melia. 2019. ATG2 transports lipids to promote autophagosome biogenesis. J. Cell Biol. 218:1787–1798. 10.1083/jcb.201811139 30952800 PMC6548141

[bib48] Wong, Y.C., and E.L. Holzbaur. 2014. Optineurin is an autophagy receptor for damaged mitochondria in parkin-mediated mitophagy that is disrupted by an ALS-linked mutation. Proc. Natl. Acad. Sci. USA. 111:E4439–E4448. 10.1073/pnas.1405752111 25294927 PMC4210283

[bib49] Wu, W., W. Tian, Z. Hu, G. Chen, L. Huang, W. Li, X. Zhang, P. Xue, C. Zhou, L. Liu, . 2014. ULK1 translocates to mitochondria and phosphorylates FUNDC1 to regulate mitophagy. EMBO Rep. 15:566–575. 10.1002/embr.201438501 24671035 PMC4210082

[bib50] Yamashita, S.I., X. Jin, K. Furukawa, M. Hamasaki, A. Nezu, H. Otera, T. Saigusa, T. Yoshimori, Y. Sakai, K. Mihara, and T. Kanki. 2016. Mitochondrial division occurs concurrently with autophagosome formation but independently of Drp1 during mitophagy. J. Cell Biol. 215:649–665. 10.1083/jcb.201605093 27903607 PMC5147001

[bib51] Yamashita, S.I., and T. Kanki. 2018. Detection of iron depletion- and hypoxia-induced mitophagy in mammalian cells. Methods Mol. Biol. 1782:315–324. 10.1007/978-1-4939-7831-1_18 29851008

[bib52] Yamashita, S.I., Y. Sugiura, Y. Matsuoka, R. Maeda, K. Inoue, K. Furukawa, T. Fukuda, D.C. Chan, and T. Kanki. 2024. Mitophagy mediated by BNIP3 and NIX protects against ferroptosis by downregulating mitochondrial reactive oxygen species. Cell Death Differ. 31:651–661. 10.1038/s41418-024-01280-y 38519771 PMC11094013

[bib53] Yang, Z., S.R. Yoshii, Y. Sakai, J. Zhang, H. Chino, R.L. Knorr, and N. Mizushima. 2024. Autophagy adaptors mediate Parkin-dependent mitophagy by forming sheet-like liquid condensates. EMBO J. 43:5613–5634. 10.1038/s44318-024-00272-5 39420095 PMC11574277

[bib54] Ylä-Anttila, P., H. Vihinen, E. Jokitalo, and E.L. Eskelinen. 2009. 3D tomography reveals connections between the phagophore and endoplasmic reticulum. Autophagy. 5:1180–1185. 10.4161/auto.5.8.10274 19855179

[bib55] Zhu, Y., S. Massen, M. Terenzio, V. Lang, S. Chen-Lindner, R. Eils, I. Novak, I. Dikic, A. Hamacher-Brady, and N.R. Brady. 2013. Modulation of serines 17 and 24 in the LC3-interacting region of Bnip3 determines pro-survival mitophagy versus apoptosis. J. Biol. Chem. 288:1099–1113. 10.1074/jbc.M112.399345 23209295 PMC3542995

