# Supplementary figures and images for: The mitophagy receptors BNIP3 and NIX mediate tight attachment and expansion of the isolation membrane to mitochondria
Source: J Cell Biol. 2025 May 13;224(7):e202408166. doi: 10.1083/jcb.202408166 (PMC12071194; doi:10.1083/jcb.202408166)

Fig. S3C

BNIP3

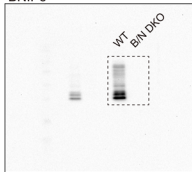

NIX

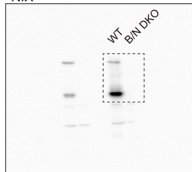

BCL2L13

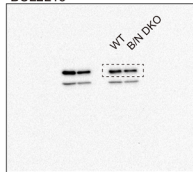

FKBP8

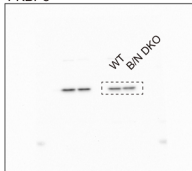

FUNDC1

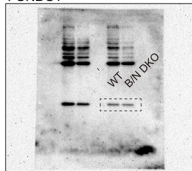

Actin

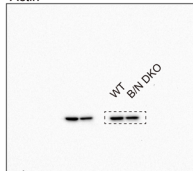

Supplement: SourceData FS3 — is the source file for Fig. S3. [file jcb_202408166_sourcedatafs3.pdf]

Fig. S5D

BNIP3

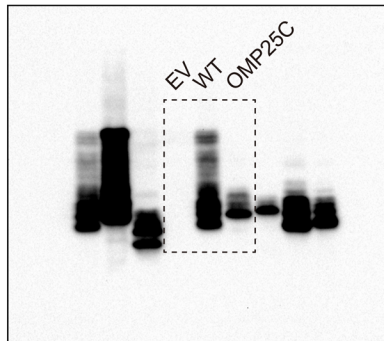

Actin for BNIP3

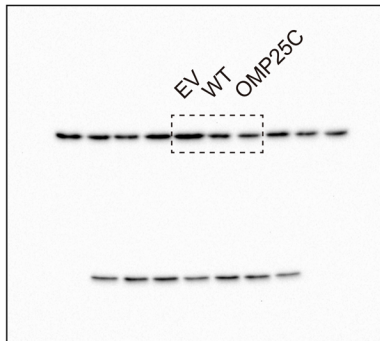

NIX

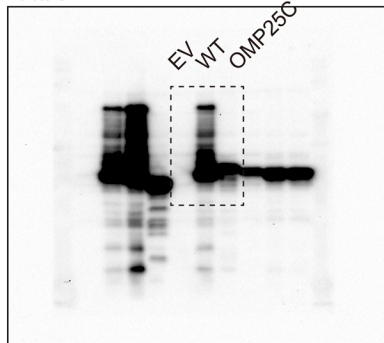

Actin for NIX

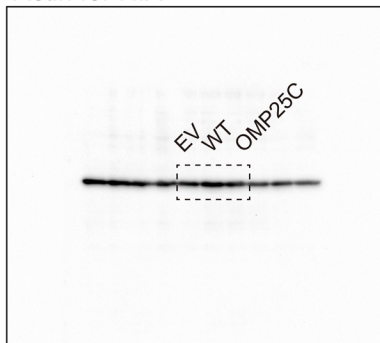

Supplement: SourceData FS5 — is the source file for Fig. S5. [file jcb_202408166_sourcedatafs5.pdf]
