# Supplementary material for: The mitophagy receptors BNIP3 and NIX mediate tight attachment and expansion of the isolation membrane to mitochondria
Source: J Cell Biol. 2025 May 13;224(7):e202408166. doi: 10.1083/jcb.202408166 (PMC12071194; doi:10.1083/jcb.202408166)
Supplement: SourceData FS4 — is the source file for Fig. S4. [file jcb_202408166_sourcedatafs4.pdf]

Fig. S4D

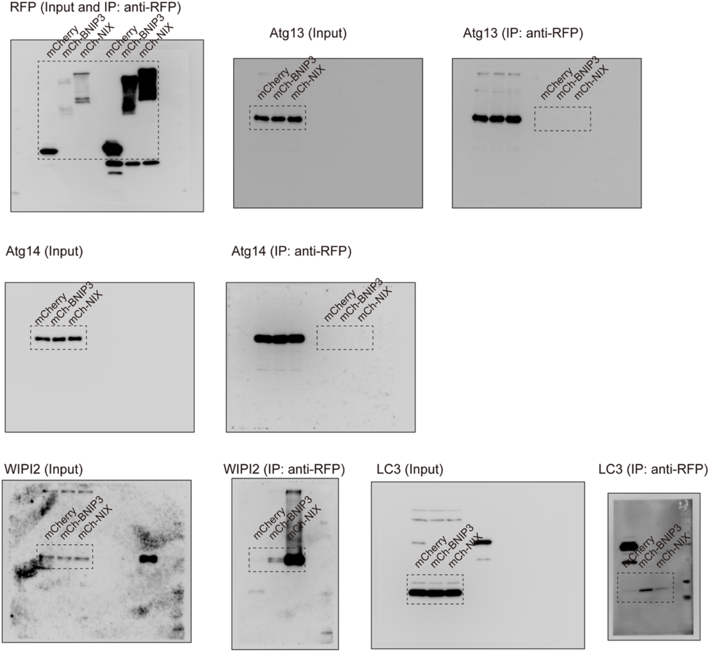

RFP (Input and IP: anti-RFP)
mCherry
mCh-BNIP3
mCh-NIX
mCherry
mCh-BNIP3
mCh-NIX
Atg13 (Input)
mCherry
mCh-BNIP3
mCh-NIX
Atg13 (IP: anti-RFP)
mCherry
mCh-BNIP3
mCh-NIX
Atg14 (Input)
mCherry
mCh-BNIP3
mCh-NIX
Atg14 (IP: anti-RFP)
mCherry
mCh-BNIP3
mCh-NIX
WIPI2 (Input)
mCherry
mCh-BNIP3
mCh-NIX
WIPI2 (IP: anti-RFP)
mCherry
mCh-BNIP3
mCh-NIX
LC3 (Input)
mCherry
mCh-BNIP3
mCh-NIX
LC3 (IP: anti-RFP)
mCherry
mCh-BNIP3
mCh-NIX
